# Palmitoylated claudin7 captured in glycolipid-enriched membrane microdomains promotes metastasis *via* associated transmembrane and cytosolic molecules

**DOI:** 10.18632/oncotarget.8928

**Published:** 2016-04-22

**Authors:** Florian Thuma, Sarah Heiler, Martina Schnölzer, Margot Zöller

**Affiliations:** ^1^ Department of Tumor Cell Biology, University Hospital of Surgery, Heidelberg, Germany; ^2^ Department of Functional Proteome Analysis, German Cancer Research Center, Heidelberg, Germany

**Keywords:** claudin7 palmitoylation, metastasis, glycolipid-enriched membrane microdomains, complex-dependent bioactivity

## Abstract

In epithelial cells claudin7 (cld7) is a major component of tight junctions, but is also recovered from glycolipid-enriched membrane microdomains (GEM). In tumor cells, too, cld7 exists in two stages. Only GEM-located cld7, which is palmitoylated, promotes metastasis. Searching for the underlying mechanism(s) revealed the following.

The metastatic capacity of the rat pancreatic adenocarcinoma cell line ASML is lost by a knockdown (kd) of cld7 and is not regained by rescuing cld7 with a mutated palmitoylation site (cld7^mPalm^). ASML-cld7^kd^ and ASML-cld7^mPalm^ cells show reduced motility and invasiveness. This is due to cld7, but not cld7^mPalm^ associating with α6β4, ezrin, uPAR and MMP14, which jointly support motility and invasion. Palmitoylated cld7 also is engaged in drug resistance by repressing Pten, allowing activation of the antiapoptotic PI3K/Akt pathway. An association of cld7^mPalm^ with the major Pten phosphorylating kinases does not restore apoptosis resistance as phosphorylated Pten is not guided towards GEM to compete with non-phosphorylated Pten. The pathway whereby palmitoylated cld7 supports expression of several EMT genes and nuclear translocation of EMT transcription factors remains to be unraveled. An association with Notch, reduced in ASML-cld7^mPalm^ cells, might be the starting point. Finally, GEM-located, palmitoylated cld7 associates with several components of vesicle transport machineries engaged in exosome biogenesis.

Taken together, prerequisites for cld7 acting as a cancer-initiating cell marker are GEM location and palmitoylation, which support a multitude of associations and integration into exosomes. The latter suggests palmitoylated cld7 contributing to message transfer via exosomes.

## INTRODUCTION

Cancer remains one of the leading causes of death [1]. This mostly is due to the capacity of malignant cells to metastasize, the unpredictable spread of tumor cells frequently setting the corner stone for curative therapy [2]. Evidence is accumulating that limitations in cancer therapy can be overcome by attacking a small population of cancer-initiating cells (CIC) that are essential for primary tumor and metastatic growth [3]. CIC are characterized by tumorigenicity, self-renewal and differentiation capacity, anchorage independent growth, longevity and drug resistance [4]. CIC are also defined by sets of surface markers [5], which were repeatedly reported to be of functional relevance [6, 7]. In gastrointestinal cancer evidence was provided that EpC acts as a CIC biomarker, but requires support by claudin7 (cld7) [8–10]. Experimental evidence pointing towards a dominance of the cld7 contribution demanded controlling the genuine cld7 activity [9].

Claudins are a family of four-pass tight junction (TJ) proteins [11–13]. The importance of clds, including cld7, was repeatedly demonstrated by targeted deletion (ko). Cld1^ko^ mice die within one day after birth due to severe defects in the barrier functions of the skin [14]. A cld7^ko^ is lethal within 10 days after birth due to destruction of the intestine [15]. The authors speculate on a missing association with integrins and a striking upregulation of MMP9 contributing to gut destruction [15]. An intestine-specific conditional cld7^ko^ mouse revealed a specific enhancement of paracellular small organic solute flux across the TJ, which included N-formyl-L-methionionyl-L-leucyl-L-phenylalanine (fMLP), a major bacterial product that initiates colonic inflammation [16].

However, clds can also be diffusely distributed in lateral membranes [17–20]. This accounts particularly for cld7 [21–23] and was first described for the localization in kidney tubuli [24]. Claudins are PKA, PKC and MLCK targets [25–29], where cld phosphorylation can prohibit integration into TJ, which is accompanied by loss of epithelial cell polarization [30–32]. We expect that multiple phosphorylation sites need to be affected, as we did not observe relocation, when mutating individual serine residues [33]. Instead, our studies confirmed claudin7 palmitoylation and partitioning into glycolipid-enriched membrane microdomains (GEM) [9, 10, 33, 34]. GEM are known to harbor palmitoylated proteins and due to the particular lipid composition to function as a scaffold creating a platform for signal transduction and, via cytoskeleton linker molecules, for reorganization of the cytoskeleton [35–38]. GEM are additionally prone for internalization [39, 40], where GEM membrane and linked cytosolic molecules are recruited into early endosomes, the GEM complexes being maintained and recovered in exosomes [41–43]. In concern about the contribution of clds to oncogenesis and tumor progression, several reports describe TJ proteins preventing or promoting tumor progression [18, 44–46]. Our data pointing towards functional importance of cld7 in tumor progression [9, 10], we want to mention particularly one report on cld7 expression in triple negative breast cancer. The bulk tumor does not express cld7-associated rab25, but expression is seen in CIC [47]. We interpret these data in the sense that cld7 and palmitoylated cld7 account for distinct, non-overlapping activities such that dependent on the cellular context, the functional engagement in TJ or in GEM are dominating. To circumvent the problem of skewed results, we transfected HEK cells with palmitoylation deficient cld7 (cld7^mPalm^), which confirmed strong enrichment only of palmitoylation-competent cld7 in GEM [33]. To elaborate palmitoylated cld7 selective activities, we here rescued a cld7^kd^ in a metastatic pancreatic tumor line with cld7^mPalm^.

EpCAM (EpC) is a CIC marker [48], frequently associated with cld7 [9, 36, 49–52]. The oncogenic and tumor progression supporting activity of EpC is due to EpC interfering with E-cadherin-mediated cell-cell adhesion via disrupting the link between α-catenin and F-actin [53] as well as by its engagement in Wnt/β-catenin signaling [54] and by controlling cell movement via down-regulation of PKC [55] and regulation of MMP7 expression [56, 57]. These activities are promoted by the cytoplasmic tail of EpC (EpICD), which forms a complex with β-catenin, FHL2 (four-and-half-LIM-only) and Lef-1, relocates to the nucleus and initiates c-myc, cyclin A and E transcription [58]. EpICD also initiates transcription of reprogramming genes like Oct4 and Nanog, which is accompanied by epithelial-mesenchymal transition (EMT) with upregulation of vimentin, Snail, Slug and downregulation of E-cadherin in a colon cancer and a hepatoma line [59]. Though not evaluated, it is tempting to speculate on a cld7 contribution as hepatocyte progenitors express EpC and cld7 [60] and in colon and pancreatic cancer, EpC is cld7-associated [50]. Under physiological conditions, too, the EpC-cld7 association appears vital. An EpC^ko^, associated with intestine destruction-promoted death within one week after birth, is due to the missing association of EpC with cld7 [61]. These findings pointed towards a concerted activity of EpC and cld7 in tumor progression, which was confirmed by a cld7^kd^ and an EpC^kd^ in a metastasizing line, which both sufficed to wave metastatic growth [9]. As EpC is one of the dominating partners of cld7, we also generated an EpC rescue with a mutation of the cld7-binding site (EpC^mAG^) to differentiate not only between non-palmitoylated and palmitoylated cld7, but also on the impact of an EpC association.

We confirm that only palmitoylated cld7 promotes tumor progression by supporting motility and invasion, GEM location-dependent activation of the PI3K/Akt pathway and upregulation of mesenchymal genes. Finally, GEM-located palmitoylated cld7 associates with vesicle transporter complexes. This might have severe consequences on exosome delivery and the communication with neighboring tumor, stroma and hematopoietic cells.

## RESULTS

Cld7 belongs to the family of TJ proteins, supposed to inhibit tumor progression. However, cld7 is also recovered outside of TJ. We provided evidence that palmitoylated cld7 is enriched in GEM, cooperating with GEM-, but not TJ-located molecules [33]. Indeed, non-palmitoylated versus palmitoylated cld7 exhibit on non-overlapping and opposing activities.

### The model

ASML is a metastasizing pancreatic adenocarcinoma [62], highly expressing cld7 and EpC. At least part of the two molecules are associated via a direct protein-protein interaction [50]. A cld7^kd^ as well as an EpC^kd^ are accompanied by loss in metastatic potential [9]. To control for the impact of cld7 palmitoylation, ASML-cld7^kd^ cells were rescued with a palmitoylation site mutated cld7 (ASML-cld7^mPalm^). A transient cld7 rescue served as control. As ASML-EpC^kd^ cells also do not metastasize, which could be due to palmitoylated cld7 supporting the generation of the cotranscription factor EpICD [33], we additionally generated an ASML-EpC^resc^ line and a rescue line, where the binding site for cld7 is mutated (ASML-EpC^mAG^). The latter allows judging, whether functional activity of palmitoylated cld7 is strictly linked to associated EpC.

**Figure 1:**
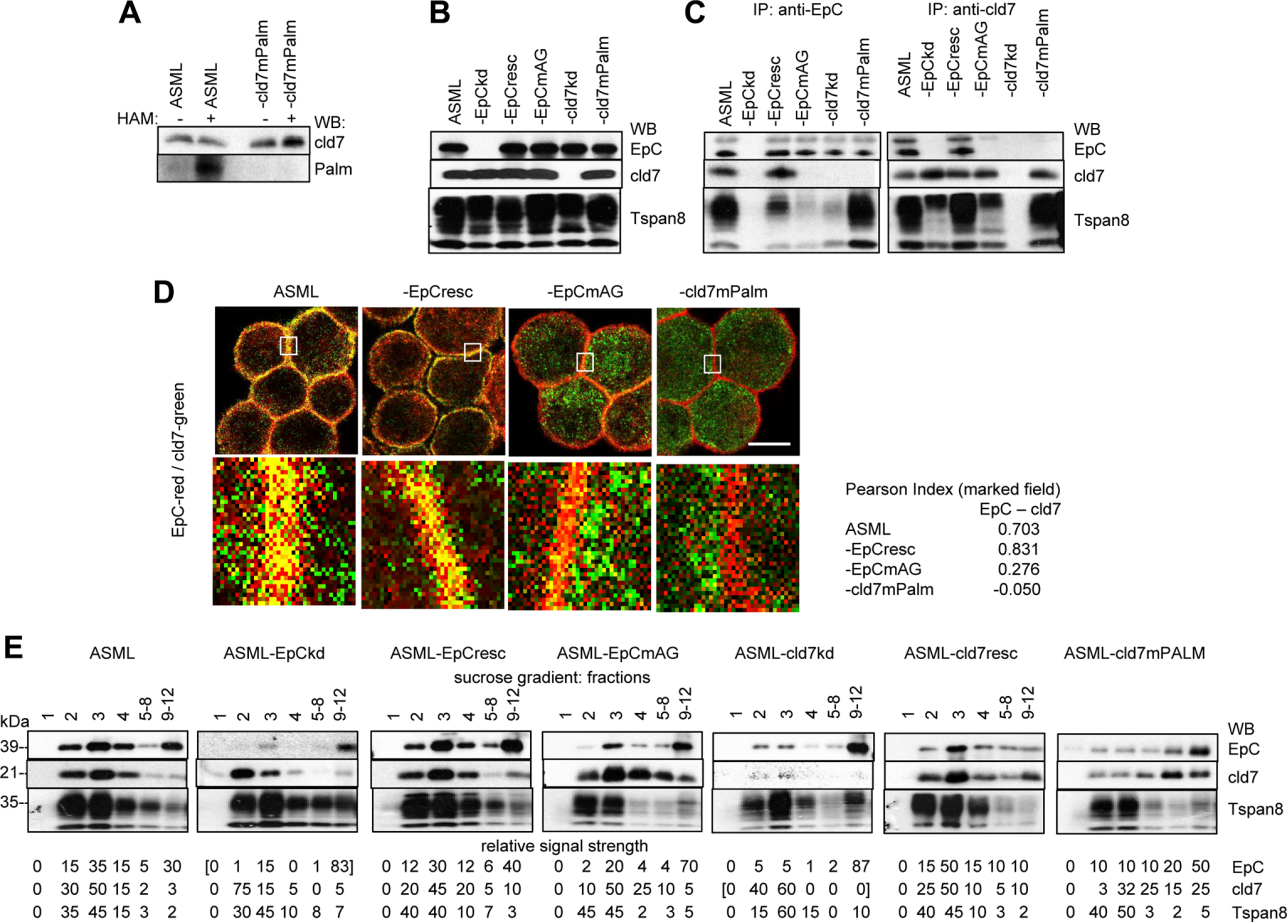
Characterization of ASML-EpC and cld7 knockdown and rescue clones ASML cells were transfected with EpC- and cld7-shRNA and cloned in selection medium. EpC expression was rescued in ASML-EpC^kd^ clones using primers for wt rescue (EpC^resc^) or point mutated (position 282 and 279) EpC (EpC^mAG^); cld7 was rescued in ASML-cld7^kd^ clones with a mutation at the palmitoylation site at AA184 and AA186 or was transiently rescued using primers for wt rescue (cld7^resc^). (**A**) Wt, kd and rescue ASML clones were lysed in the presence of N-ethylmaleimide (NEM) to irreversibly block unmodified thiol groups. After incubation with HAM buffer for unmasking palmitoylated cysteine thiol groups, samples where incubated in biotin-BMCC for selective labeling of palmitoylated cysteines. Samples were blotted with streptavidin-HRP and after stripping with anti-cld7; (**B**) lysates of wt, kd and rescue ASML clones (one representative clone was selected) were separated by SDS-PAGE and blotted with anti-EpC (D5.7), anti-cld7 and anti-Tspan8 (D6.1, control); (**C**) lysates of wt, kd and rescue ASML clones were precipitated with anti-EpC or anti-cld7. After SDS-PAGE, precipitates were blotted with anti-EpC, anti-cld7 or anti-Tspan8; (**D**) wt and rescue ASML clones were stained with anti-EpC(red) and anti-cld7 (green); staining was evaluated by confocal microscopy, digital overlays (scale bar: 10 μM). The indicated area (white square) was amplified 10-fold for better discrimination. The Pearson correlation coefficiency is shown for the encircled membrane area; (**E**) lysates of wt, kd and rescue ASML clones were separated according to density by sucrose gradient centrifugation; 1ml fractions were collected and fractions 5–8 and 9–12 were pooled. After SDS-PAGE, fractions were blotted with anti-EpC, anti-cld7 and anti-Tspan8 (GEM control). EpC and cld7 were efficiently downregulated in kd clones and were recovered in rescue clones. Mutation of the palmitoylation site at AA184 and AA186 prevented cld7 palmitoylation. Cld7 palmitoylation strongly facilitates the association with EpC, the cld7-EpC complex being enriched in GEM.

Cld7 has two palmitoylation sites. A palmitoylation assay revealed that mutating AA184 and AA186 prevents cld7 palmitoylation indicating that predominantly the cld7 C-terminal tail is palmitoylated (Figure 1A). ASML-cld7^mPalm^ express cld7 at a comparable level to ASML^wt^ cells. This also accounts for EpC expression in ASML-EpC^resc^ and ASML-EpC^mAG^ (Figure 1B). Furthermore, cld7 co-immunoprecipitates with EpC, but not with EpC^mAG^. Cld7^mPalm^ does not coimmunoprecipitate with EpC. Both EpC and cld7 coimmunoprecipitate with the tetraspanin Tspan8, but coimmunoprecipitation with EpC^mAG^ is impaired (Figure 1C). Confocal microscopy confirmed strong colocalization of cld7 with EpC in ASML^wt^ and ASML-EpC^resc^ cells, but poor colocalization in ASML-EpC^mAG^ and ASML-cld7^mPalm^ cells (Figure 1D). Furthermore, in ASML^wt^ and -cld7^resc^ cells cld7 and EpC are enriched in light density (GEM) fractions, but cld7^mPalm^ is shifted towards heavier fractions. Recovery of EpC in light density fractions depends on the association with (palmitoylation competent) cld7. EpC is poorly recovered in light density fractions of ASML-cld7^kd^ and -EpC^mAG^ lysates and is not rescued into light density fractions in ASML-cld7^mPalm^ lysates. Recovery of the constitutively GEM-located tetraspanin Tspan8 is not affected by the cld7^kd^ or cld7^mPalm^ (Figure 1E).

Taken together, in ASML cells, which do not form TJ, cld7 is palmitoylated and enriched in GEM. The association with EpC is promoted by cld7 palmitoylation, but is not completely abolished in the absence of (palmitoylated) cld7, possibly due to EpC associating also with other palmitoylated GEM-located molecules, like e.g. Tspan8.

### Anchorage independence and metastasis formation require palmitoylation-competent cld7

**Figure 2:**
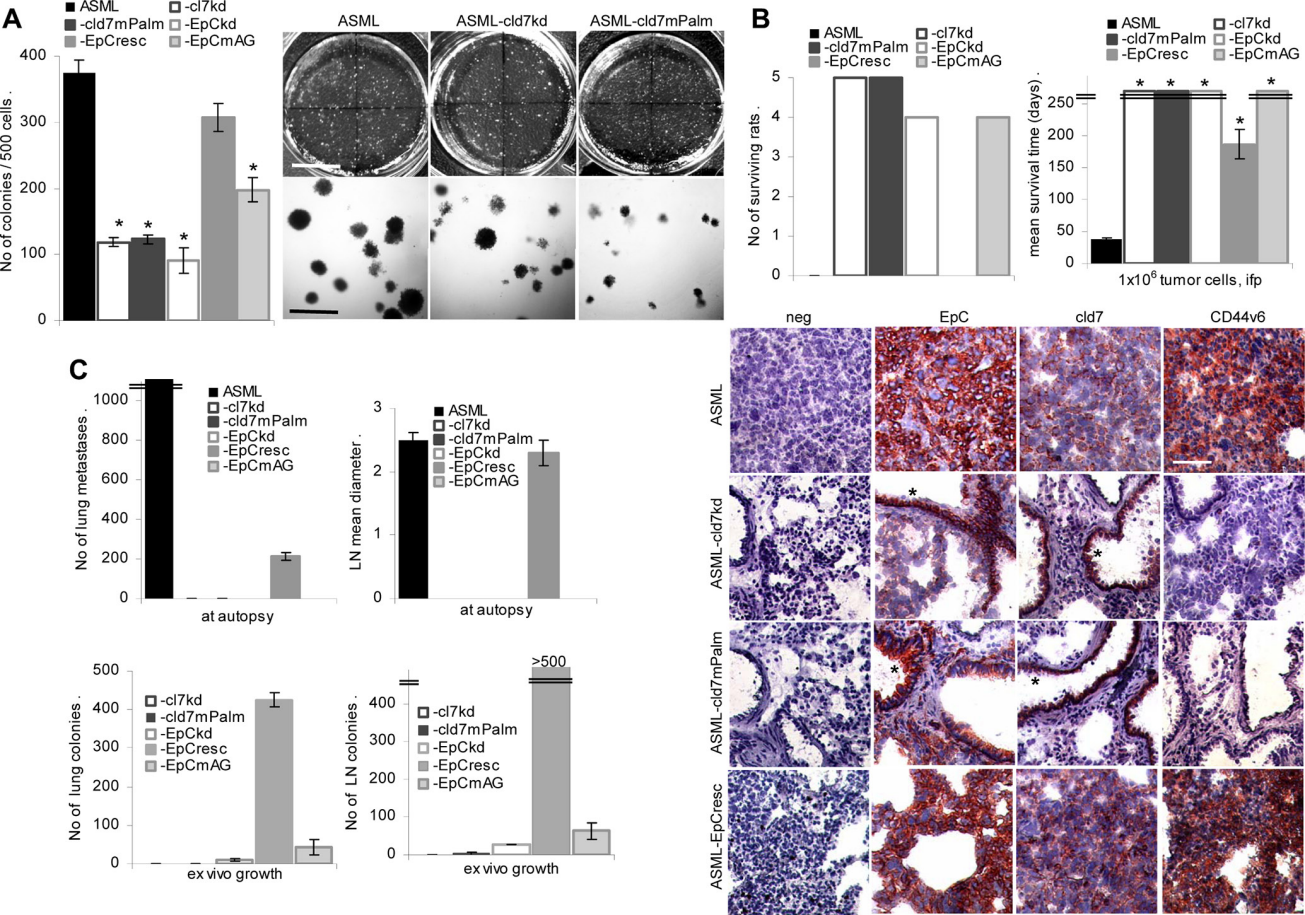
Palmitoylated cld7 supports anchorage independent growth and metastasis formation (**A**) Wt, kd and rescue ASML cells (500) were seeded in soft agar in 10 cm Petri-dishes. Colonies were counted after 14 d. The mean number ± SD (triplicates) of colonies and representative examples are shown. (B, C) BDX rats received 1 × 10^6^ wt, kd or rescue ASML cells, ifp. (**B**) The number of surviving rats and the mean survival time are shown; (**C**) the number of visible lung metastases, the mean draining lymph node diameter, the number of tumor cell colonies growing in suspended lung and LN tissue was evaluated after 4 wk. (D) Shock frozen lung sections from ASML wt, -cld7^kd^, cld7^mPalm^ and -EpC^resc^ bearing rats were stained with anti-EpC, -cld7 and -CD44v6 (scale bar: 120 μm). Tumor cells (EpC^+^, cld7^+^, CD44v6^+^) are only seen in the lung of ASML^wt^ and -EpC^resc^ bearing rats. In the lungs of ASML-cld7^kd^ and -cld7^mPalm^ bearing rats only bronchiolar epithelium (*) is stained by anti-EpC and anti-cld7. Anchorage-independent growth and metastasis are severely impaired in both kd clones, but are regained in ASML-EpC^resc^ and partly ASML-EpC^mAG^, but not in -cld7^mPalm^ cells, indicating only palmitoylated cld7 supporting metastasis.

Anchorage independence and tumor progression are central features of CIC. Soft agar colony formation of ASML-cld7^kd^ and ASML-EpC^kd^ cells is strongly decreased, but is largely regained in ASML-EpC^resc^ and partly in ASML-EpC^mAG^ cells. Soft agar colony formation is not restored in ASML-cld7^mPalm^ cells, which start to form small clusters, but die after 1 wk of culture (Figure 2A).

ASML-cld7^kd^ cells completely lost the capacity to metastasize via the lymphatic system and metastatic capacity is not rescued in ASML-cld7^mPalm^ cells. None of the rats developed visible metastasis. Very few tumor cell colonies grew in *ex vivo* cultured lymph node and none in lung suspensions. Instead, ASML-EpC^resc^ cells develop lymph node metastases and a limited number of lung metastases after intrafootpad application. Although with a significant delay, ASML-EpC^resc^ bearing rats become moribund after 154–215 days mostly due to the metastatic lymph node burden. Few ASML-EpC^mAG^ cells were recovered in lymph nodes and lung in *ex vivo* cultures, but did not form visible metastases. Immunohistology confirmed that ASML and ASML-EpC^resc^ cells displaced the lung tissue with only EpC^+^/cld7^+^/CD44v6^+^ tumor cells being seen in most sections. Instead, no tumor nodules were seen in the lung of rats that received ASML-cld7^kd^ or ASML-cld7^mPalm^ cells, only bronchiolar epithelial cells being stained by anti-EpC and anti-cld7 (Figure 2B, 2C).

Thus, palmitoylated cld7 is indispensable for ASML metastasis formation. There are 3 major, mutually not exclusive features, whereby palmitoylated cld7 could support the metastasis process. (i) Palmitoylated cld7 promotes tumor cell motility by associating with integrins and the cytoskeleton and/or by cooperating with proteases to create space for metastases; (ii) palmitoylated cld7 is engaged in apoptosis resistance and (iii) EMT.

### Palmitoylated cld7 and motility

**Figure 3:**
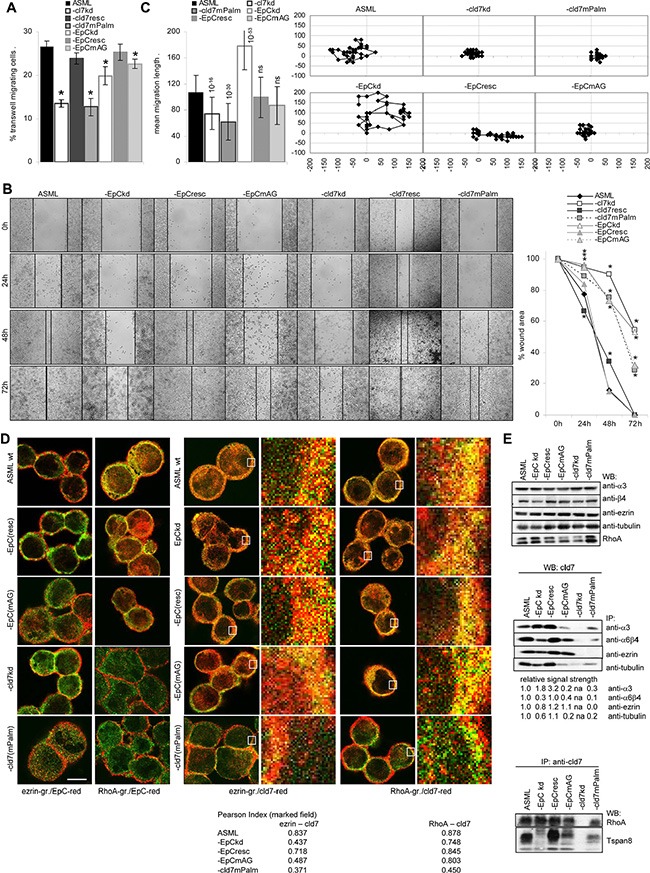
The impact of palmitoylated cld7 on cell motility (**A**) Wt, kd and rescue ASML cells (2 × 10^4^ in RPMI/1% BSA) were seeded in the upper part of a Boyden chamber; the lower part, separated by a 0.8 μm pore size membrane contained RPMI/20% FCS. Recovery of cells on the lower membrane site was evaluated after 16 h by crystal violet staining. The percent ± SD of migrating cells compared to the total input are shown. (**B**) Wt, kd and rescue ASML cells were seeded in 24-well plates. When cultures reached a subconfluent stage, the monolayer was scratched with a pipette tip. Wound healing was followed for 72 h. Examples (scale bar: 250 μm) and the mean percent ± SD of the wound area compared to the 0 time point are shown. (**C**) Cells as above were seeded in 6-well plates coated with LN111. Pictures were taken every 20 min for 24 h. Migration of 20 individual cells was recorded. An example of migration of a single cell as well as the mean migration ± SD of 20 cells/well is presented. (A–C) Significant differences as compared to ASML^wt^ cells: *. (**D**) Wt, kd and rescue ASML cells were stained with anti-ezrin (green) or anti-RhoA (green) and anti-EpC (red) or anti-cld7 (red). Staining was evaluated by confocal microscopy; digital overlays of staining are shown (scale bar: 10 μm). The indicated area (white square) was amplified 10-fold for better discrimination. The Pearson correlation coefficient is shown for the encircled membrane area. (**E**) Lysates of cells as above were precipitated with anti-α3, -α6β4 (B5.5), -ezrin and -tubulin and were blotted with anti-cld7 or were precipitated with anti-cld7 and blotted with -RhoA and -Tspan8. The relative signal strength of cld7 precipitates is indicated. The strength of the cld7 signal in ASML wt was arbitrarily set as 1.0. WB of α3, β4, ezrin, tubulin and Rho are included as controls. Migration of ASML-cld7^kd^ and -cld7^mPalm^, but not of -cld7^resc^ cells is severely reduced. Impaired migration is accompanied by reduced association of cld7^mPalm^ with ezrin, and, less pronounced, α3, α6β4, tubulin, RhoA and Tspan8. In the absence of (palmitoylated) cld7, EpC does poorly colocalize/associate with RhoA.

ASML cells do not grow locally, the capacity to leave the injection site and to reach the first lymph node station becoming vital. Transwell migration and wound healing of ASML-cld7^kd^ and -EpC^kd^ cells is significantly reduced. It is restored in ASML-cld7^resc^ and -EpC^resc^ cells, but not in ASML-cld7^mPalm^ and -EpC^mAG^ cells (Figure 3A, 3B). In transwell migration the cld7^kd^ exerted a stronger effect than the EpC^kd^, which was controlled for the migration of individual cells by videomicroscopy. Distinct to the reduced migration of ASML-cld7^kd^ and -cld7^mPalm^ cells, migration of single ASML-EpC^kd^ cells was increased and migration of -EpC^mAG^ was not affected (Figure 3C). This finding indicates that cld7 actively promotes motility, whereas “free” EpC hampers motility, though to a minor degree.

In order to define the mechanism(s) underlying palmitoylated cld7-promoted motility, we searched by mass spectrometry for proteins preferentially co-immunoprecipitating after mild lysis, not to destroy GEM complexes, with cld7 and/or cld7^mPalm^. ASML^wt^ were precipitated with anti-EpC and anti-cld7. ASML-EpC^kd^, -EpC^mAG^ and -cld7^mPalm^lysates were precipitated with anti-cld7. A considerable number of cytoskeletal proteins associate with EpC and cld7. While integrins and some tetraspanins preferentially coimmunoprecipitate with EpC (Supplementary Table S1A), the association requires palmitoylation-competent cld7. This is demonstrated for α6β4 and Tspan8 that do not or poorly colocalize with EpC in ASML-EpC^mAG^ and -cld7^mPalm^ cells and most poorly with cld7 in ASML-cld7^mPalm^ cells (Supplementary Figure S1). Α6β4 and Tspan8 also poorly coimmunoprecipitate with cld7 in ASML-cld7^mPalm^ lysates. Coimmunoprecipitation of α3 with cld7 is less severely affected in ASML-cld7^mPalm^ cells (Figure 3E). Furthermore, the cytoskeletal linker proteins actinin, moesin and RhoA, which are engaged in actin cytoskeleton organization, preferentially associate with palmitoylation-competent cld7. On the contrary, cytoskeletal keratins and myosin associate with EpC and cld7, but more readily with non-palmitoylated cld7 (Supplementary Table S1A). Confocal microscopy confirmed strongly reduced colocalization of ezrin and RhoA with cld7^mPalm^. EpC poorly colocalizes with ezrin and RhoA in ASML-EpC^mAG^ and -cld7^mPalm^ (Figure 3D). WB of immunoprecipitates confirmed the association of cld7 with ezrin, tubulin and RhoA. However, ezrin does not coimmunoprecipitate with cld7^mPalm^ and coimmunoprecipitation of tubulin and RhoA with cld7 is strongly reduced in ASML-cld7^mPalm^ precipitates. Finally, cld7 poorly coimmunoprecipitates with α3 and tubulin in ASML-EpC^mAG^ and with Tspan8 in ASML-EpC^kd^ and -EpC^mAG^ cells, which indicates a contribution of cld7-associated EpC to complex formation (Figure 3E).

Taken together, multiple associations between cld7, actin linker and actin (re)organizing proteins well explain the reduced motility of ASML-cld7^kd^ cells. Fittingly, palmitoylation-competent cld7 poorly associates with keratins.

### Palmitoylated cld7 and invasiveness

**Figure 4:**
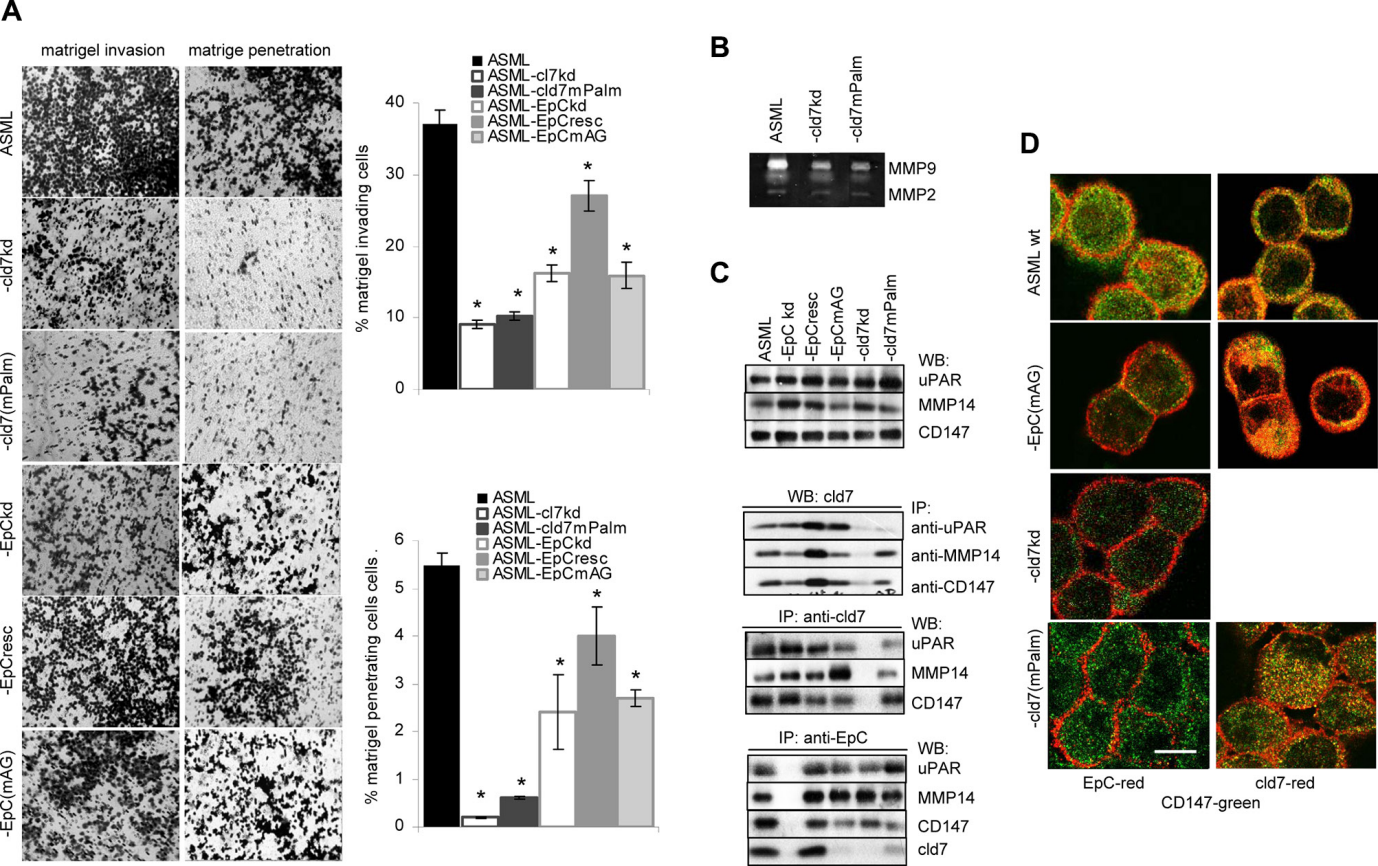
Palmitoylated claudin7 supports invasiveness and protease activity (**A**) Wt, kd and rescue ASML cells were seeded on matrigel. Matrigel invasion and penetration was evaluated after 16 h incubation. The mean percent ± SD of invading and penetrating cells and representative examples are shown. (**B**) Zymography of culture supernatants of ASML^wt^, -cld7^kd^ and -cld7^mPalm^ cells; MMP2 and MMP9 bands are indicated. (**C**) Lysates from cells as in (A) were precipitated with anti-CD147 (EMMPRIN), anti-uPAR and anti-MMP14 and were blotted with anti-cld7 or were precipitated with anti-cld7 or anti-EpC and blotted with anti-uPAR and anti-CD147. WB of lysates with anti-uPAR, -MMP14 and -CD147 are included as controls. (**D**) Cells as above were stained with anti-CD147 (green) and either anti-EpC (red) or anti-cld7 (red). Digital overlays are shown (scale bar: 10 μm). ASML-cld7^kd^ cells are poorly invasive and invasiveness is not restored in ASML-cld7^mPalm^ cells. This also accounts for reduced MMP9 activity and fits to reduced coimmunoprecipitation of cld7^mPalm^ with MMP14 and uPAR. EpC has a weaker impact on invasiveness and MMP activity.

Matrigel invasion and penetration of ASML-cld7^kd^ cells is strongly reduced and is not rescued in ASML-cld7^mPalm^ cells. Invasion and penetration of ASML-EpC^kd^ and -EpC^mAG^ cells is also reduced, although less efficiently (Figure 4A). However, the protease profile of ASML cells is not significantly altered in ASML-cld7^kd^ and ASML-EpC^kd^ and rescue clones (Supplementary Figure S2). Instead, particularly MMP9 activity (zymography) is strongly reduced in ASML-cld7^kd^ and ASML-cld7^mPalm^ cells (Figure 4B). Co-immunoprecipitation (mass spectrometric analysis, data not shown), revealed an association with uPAR and CD147 (basigin), the latter recruiting proteases from neighboring cells. These associations and an association with MMP14 were confirmed by WB after co-immunoprecipitation. The association with uPAR depended on cld7 palmitoylation, whereas the association with MMP14 and CD147 was also seen in ASML-cld7^mPalm^ cells. The latter finding is supported by strong colocalization of CD147 with cld7^mPalm^ (Figure 4C, 4D).

We demonstrated before that several proteases associate with GEM-located tetraspanins and also with CD44v6 [63, 64]. As invasiveness was not rescued in ASML-cld7^mPalm^, we suggest that the contribution of cld7 is linked to the integration of palmitoylated cld7 into GEM, rather than to a direct impact of cld7 on protease activity.

### Apoptosis resistance, cld7 and PTEN

**Figure 5:**
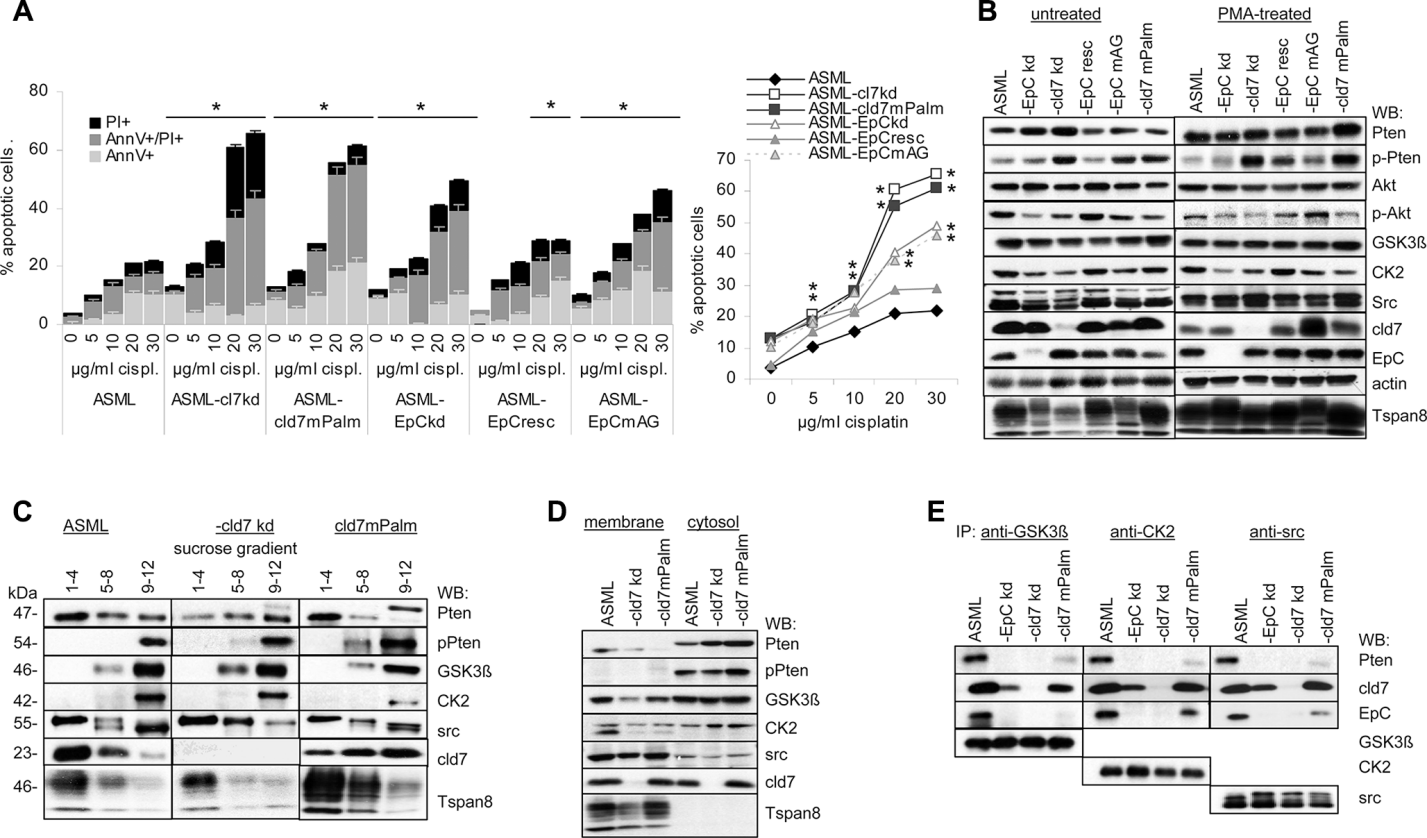
Cld7 and apoptosis resistance (**A**) Wt, kd and rescue ASML cells were cultured in the presence of increasing amounts of cisplatin. Flow cytometry analysis of the percent of AnnV+, AnnV+PI+ and PI+ cells; mean ± SD (triplicates) are shown, significant differences to wt cells: *. (**B**) WB of Akt, Pten, pPten and Pten phosphorylating kinases in untreated and PMA-treated wt, kd and rescue ASML cells; (**C**) sucrose gradient fractions of the lysates as above and WB with anti-Pten, -pPten, -GSK3β, -CK2, -src, -cld7 and -Tspan8; (**D**) Membrane and cytosol lysates from cells as above were blotted with anti-GSK3β, -CK2, -src, -pPten and -cld7. (**E**) WB with anti-Pten, -cld7, -EpC, -GSK3β, -CK2 and -src after precipitation with anti-GSK3β, anti-CK2 and anti-src. Drug resistance of ASML-cld7^kd^ cells is severely impaired and not rescued in ASML-cld7^mPalm^ cells. Upregulation of Pten in PMA-treated ASML-cld7^kd^ and -cld7^mPalm^ cells indicates Pten repression only by palmitoylation-competent cld7. Concomitant pronounced cytoplasmic Pten phosphorylation does not contribute rescuing apoptosis resistance.

High drug resistance of ASML cells is strongly reduced in ASML-cld7^kd^ and is not rescued in -cld7^mPalm^ cells. Drug resistance of ASML-EpC^kd^ cells also is reduced, though less severely. Drug resistance is re-established in ASML-EpC^resc^ and partially in -EpC^mAG^ cells. This accounted for AnnV/PI staining (apoptosis), mitochondrial integrity (MTT assay) and proliferation (^3^H-thymidine incorporation) (Figure 5A, Supplementary Figure S3A).

In the absence of stress, expression of proteins engaged in receptor-mediated apoptosis or the mitochondrial pathway of apoptosis is unaltered. However, cisplatin-treated ASML-cld7^kd^ and -cld7^mPalm^ cells show increased levels of activated Casp3 and cleaved Casp9 (Supplementary Figure S3B, S3C), reduced phosphorylated PI3K, Akt and BAD as well as Bcl2 and BclXl recovery. Slightly upregulated BID, BAK, BAX and Smac/Diablo expression is not dependent on cld7 palmitoylation (Supplementary Figure S3D). mTOR is downregulated in ASML-cld7^kd^, -cld7^mPalm^, -EpC^kd^ and -EpC^mAG^. Compared to ASML cells, Pten expression is upregulated in ASML-cld7^kd^ and ASML-cld7^mPalm^ cells. However, Pten phosphorylation is also upregulated (Supplementary Figure S3E). The flow-cytometry analysis was confirmed by a signaling protein array and/or by WB (Supplementary Figure S3D–S3F, Figure 5B). Pronounced Pten phosphorylation in ASML-cld7^kd^ and -cld7^mPalm^ opposed expectation, as the cells did not regain apoptosis resistance. Furthermore, expression of GSK3β, CK2 and src, known to be engaged in Pten phosphorylation [65], is not (GSK3β, CK2) or only slightly (src) upregulated in ASML-cld7^kd^ and -cld7^mPalm^ cells (Figure 5B). Mass spectrometry analysis of signaling molecules co-immunoprecipitating with cld7 did not provide hints towards a special reduction of serine threonine kinases co-immunoprecipitating with cld7 in ASML-cld7^mPalm^ lysates (Supplementary Table S1B). Thus, we speculated that by exclusion of palmitoylation-deficient cld7 from GEM, pronounced Pten phosphorylation does not rescue PI3K/Akt pathway activation. WB of pooled sucrose gradient fractions indicated that only src, but not GSK3βand CK2 is recovered in light density fractions. Phosphorylated Pten also is not recovered in light density fractions (Figure 5C). A WB of membrane and cytosolic lysates confirmed recovery of pPten exclusively in the cytosol, the strongest signal being seen in the ASML-cld7^mPalm^ cytosol. CK2 and GSK3β were preferentially recovered in the cytosol, src was enriched in the membrane fraction, cld7 was recovered in the membrane and the cytosolic fraction, whereas Tspan8 was exclusively recovered in the membrane fraction (Figure 5D). Thus, an association of GSK3β, CK2 and src with cytoplasmic cld7^mPalm^ could account for pronounced Pten phosphorylation. Indeed, cld7^mPalm^ coimmunoprecipitates with GSK3β, CK2 and src, which also, albeit very weakly coimmunoprecipitate with Pten (Figure 5E).

The data confirm a dominating role of palmitoylation-competent cld7 in drug resistance due to Pten repression. Cytoplasmic Pten phosphorylation via cld7^mPalm^ recruited GSK3β, CK2 and src obviously does not suffice to restore apoptosis resistance.

### Palmitoylated cld7 and EMT

**Figure 6:**
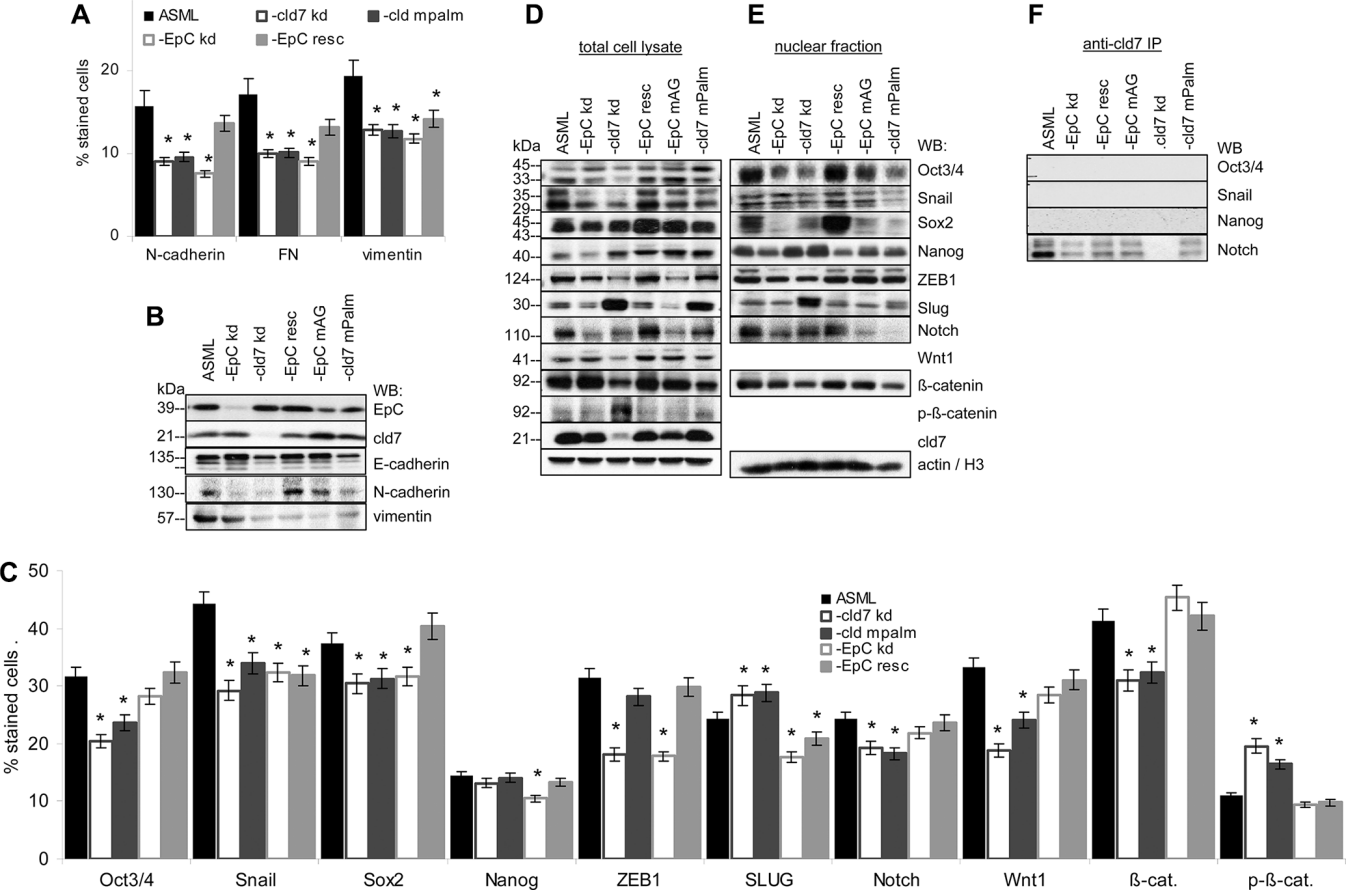
Cld7 palmitoylation and EMT gene expression (**A, C**) Wt, kd and rescue ASML cells were stained with antibodies against EMT markers, EMT-related signaling molecules and transcription factors; the mean percent ± SD of stained cells is shown. Significant differences to ASML^wt^ cells are indicated by *. (**B, D**) Total cell lysates and (**E**) the nuclear fraction of the cells as above were separated by SDS-PAGE and blotted with the indicated antibodies; (**F**) Lysates were precipitated with anti-cld7 and blotted with anti-Oct3/4, -Snail, -Nanog and -Notch. A cld7^kd^ severely affects EMT signaling molecules and transcription factors, with the exception of Slug. Reduced recovery accounts particularly for the nuclear fraction. N-cadherin, FN and vimentin expression is reduced. These changes are not reverted in ASML-cld7^mPalm^ cells. Only an EpC^kd^ is associated with E-cadherin upregulation.

Metastasis formation requires EMT. The metastasis suppressor E-cadherin is slightly upregulated in ASML-EpC^kd^, but not in ASML-cld7^kd^ cells. However, EMT-associated N-cadherin, fibronectin (FN) and vimentin expression is reduced in ASML-cld7^kd^ and ASML-EpC^kd^ cells. Expression of N-cadherin and FN is rescued in ASML-EpC^resc^, but not or less efficiently in ASML-cld7^mPalm^ cells (Figure 6A, 6B). Reduced Oct3/4, Snail, Sox2, Wnt1, Notch and β-catenin in ASML-cld7^kd^ cells also is not rescued in ASML-cld7^mPalm^ cells. Instead, p-β-catenin expression is higher in ASML-cld7^kd^ and slightly higher in -cld7^mPalm^ cells (flow-cytometry) and lysates (WB). Recovery of Oct3/4, Snail, Sox2 as well as of Notch and β-catenin also is reduced in ASML-cld7^kd^ and ASML-cld7^mPalm^ nuclei. ZEB1 is downregulated only in ASML-EpC^kd^ and -cld7^kd^ lysates. Unexpectedly, Slug expression is increased in ASML-cld7^kd^ and -cld7^mPalm^ cells and lysates (Figure 6C–6E). Cld7 does not associate with Oct, Snail and Nanog, but coimmunoprecipitates with Notch, coimmunoprecipitation being reduced in ASML-EpC^kd^, -EpC^mAG^ and -cld7^mPalm^ lysates (Figure 6F).

Taken together, palmitoylated cld7 contributes to expression of several EMT-related proteins and transcription factors and hampers β-catenin phosphorylation. The association of palmitoylated cld7 with Notch might be the initial trigger for altered EMT gene expression.

### A dominating role of GEM located cld7 in vesicle transport

Metastasis formation is supported by exosomes [66]. Though we focused on cell inherent activities of GEM-located cld7, the strong engagement of palmitoylation-competent cld7 in intracellular vesicle traffic demands mentioning.

Besides co-immunoprecipitating with adhesion molecules (Supplementary Table S1A) and mostly cytoplasmic signaling molecules (Supplementary Table S1B), cld7 also coimmunoprecipitates with soluble carrier and transporter proteins, some of which (aldose reductase, Na,K-ATPase, TM9SF2, transportin and lactadherin 2) preferentially co-immunoprecipitate with cld7^mPalm^ (Supplementary Table S1C).

**Figure 7:**
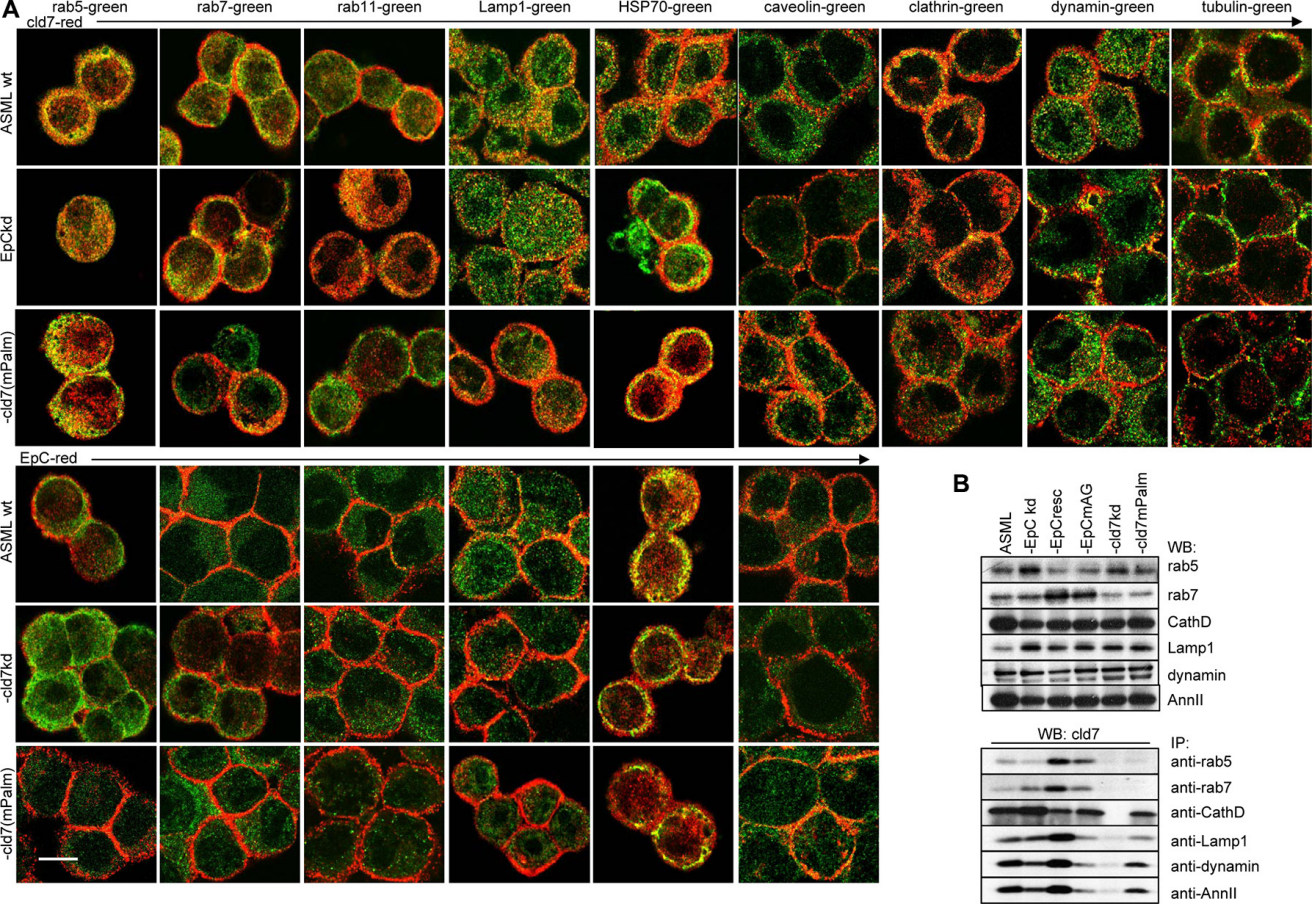
The linkage between palmitoylated cld7, vesicle formation and vesicle transporter proteins (**A**) Colocalization of cld7 (red) and EpC (red) with vesicle formation and vesicle transport associated proteins (green) in wt, EpC^kd^, cld7^kd^ and cld7^mPalm^ ASML cells. Digital overlays of confocal microscopy images are shown (scale bar: 10 μm); (**B**) WB and coimmunoprecipitation of cld7 with vesicle formation and transport proteins in wt, kd and rescue ASML cells. Only palmitoylation-competent cld7 colocalizes and coimmunoprecipitates with molecules engaged in GEM-supported vesicle formation and vesicle transport. Instead, palmitoylation-competent cld7 does not colocalize with caveolin.

GEM, including tetraspanin-enriched membrane microdomains (TEM) are prone for internalization [39, 40]. The internalization complex is maintained during intracellular vesicle traffic and vesicle exocytosis [67]. Recruitment of palmitoylation-competent cld7 and cld7-associated EpC into GEM was already demonstrated (Figure 1) as well as the GEM-located cld7 association with integrins and ARP2/3 complex components that are engaged in early endosome formation (Supplementary Table S1A). In addition, chaperons, which are enriched in exosomes, are most abundantly recovered in EpC and cld7 co-immunoprecipitates, where 6 of 28 did not or poorly associate with cld7^mPalm^ (Supplementary Table S1D). Palmitoylation-competent cld7 also associates with several transporter complexes and vesicle transport-associated molecules, particularly myoferlin, rab25 and Sec31a. Several additional rab proteins associated with cld7 independent of palmitoylation. On the opposite, only palmitoylation-deficient cld7 associated with caveolin (Supplementary Table S1E). Mostly palmitoylation-independent, cld7 associated with vesicle transporter complexes, like coatomer complexes, dynein, Vamp family proteins (Supplementary Table S1F) and tubulins (Supplementary Table S1A). Finally, cld7 abundantly associates with proteases of the proteasome complex. These associations partly depend on cld7 palmitoylation; the association with the ribophorin protease complex being only seen with palmitoylation-competent cld7 (Supplementary Table S1G). Colocalization with cld7/palmitoylated cld7 was confirmed for the vesicle transporters rab5, rab7, rab11, Lamp1 as well as for HSP70, the membrane coat clathrin, the internalization complex associated dynamin and the cytoskeletal linker protein tubulin. Notably, caveolin does not colocalize with palmitoylation-competent cld7 (Figure 7A). WB confirmed cld7 palmitoylation-dependent coimmunoprecipitation for rab5 and rab7. The association with Lamp1, CathepsinD, dynamin and AnnII was only partially cld7 palmitoylation-dependent (Figure 7B).

**Figure 8:**
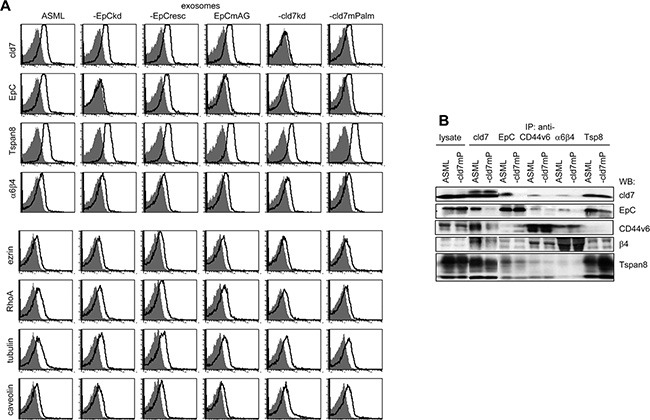
Recovery of cld7 and associated molecules in exosomes (**A**) Flow cytometry of latex beads bound wt, kd and rescue ASML exosomes stained with anti-cld7, -EpC, -Tspan8, -α6β4, -ezrin, -RhoA, -tubulin and -caveolin. Overlays of the negative control and stained samples are shown. (**B**) Lysates of ASML^wt^, and -cld7^mPalm^ exosomes were precipitated with anti-cld7, -EpC, -CD44v6, -Tspan8 or -α6β4. Dissolved precipitates were separated by SDS-PAGE and were blotted with the indicated antibodies. The WB of exosome lysates is included as control. ASML^wt^ exosomes stain brightly with anti-Tspan8, -EpC, and -cld7 and distinctly with anti-α6β4, -ezrin, -RhoA and -tubulin. ASML-cld7^mPalm^ exosomes show reduced cld7^+^, -EpC^+^ and RhoA^+^, but more caveolin^+^ exosomes. Exosome lysate coimmunoprecipitation confirmed reduced recovery of EpC, CD44v6, α6β4 and Tspan8 in anti-cld7 precipitates of ASML-cld7^mPalm^ than ASML^wt^ exosomes.

Flow cytometry confirmed high cld7, EpC, Tspan8 and distinct α6β4, ezrin, tubulin, RhoA and caveolin expression in ASML exosomes. However, the percentage of cld7^+^, EpC^+^ and RhoA^+^ exosomes was reduced, whereas the percentage of caveolin^+^ exosomes was increased in ASML-cld7^mPalm^ exosomes (Figure 8A). Coimmunoprecipitation of exosome lysates confirmed the distinct composition of palmitoylation-competent versus -deficient cld7 exosomes. Cld7 coimmunoprecipitated with the CIC markers EpC, α6β4 and Tspan8 in ASML^wt^ exosomes. Coimmunoprecipitation of EpC, CD44v6, β4 and Tspan8 was strongly reduced in ASML-cld7^mPalm^ exosomes. Also, anti-EpC, anti-CD44v6 and anti-α6β4 did not coimmunoprecipitate cld7 in ASML-cld7^mPalm^, but coimmunoprecipitation of anti-CD44v6 with anti-α6β4 and vice versa was not affected in ASML-cld7^mPalm^ (Figure 8B).

Taken together, (i) solute carrier expression is affected by a cld7^kd^; (ii) abundant associations of cld7 with stress response modulators (chaperones) could be important with respect to apoptosis resistance; (iii) the unexpectedly strong association of palmitoylated cld7 with the vesicle transporter machinery, including the deviation from the proteasome, highlights cld7 as a so far unrecognized exosome organizer. This was confirmed by the distinct composition of ASML versus ASML-cld7^mPalm^ exosomes, which also displayed different association profiles. A central role of palmitoylated cld7 in vesicle traffic and exosome biogenesis could well be a major factor in the contribution of cld7 to the CIC phenotype.

In brief, distinct to non-palmitoylated cld7 that in epithelial cells acts as a TJ protein, palmitoylated cld7 is GEM-associated and contributes in this particular environment to tumor cell motility, invasiveness, EMT and exosome biogenesis.

## DISCUSSION

Claudins were first described as TJ components that are engaged in sealing, formation of ion channels and organization of paracellular small organic solute flux [16, 68]. However, claudins are also found outside of TJ [17–24], where their functions are still disputed. We described that in tumor cells palmitoylated cld7 is recovered in GEM [9], special microdomains serving as signaling platforms [35–38] and being prone for internalization [39–41]. There was evidence that GEM-located cld7 supports motility [33] and apoptosis resistance [9]. Finally, GEM-located cld7 is associated with EpC [9, 33], the GEM-located cld7-EpC complex promoting EMT [9, 10]. Starting from this point, we evaluated the importance of cld7 palmitoylation on CIC activities in a rat metastasizing tumor model, where an EpC^kd^ was rescued with a point mutated EpC that cannot bind cld7 (EpC^mAG^). A cld7^kd^ was rescued with a mutation in the C-terminal palmitoylation site (cld7^mPalm^) that suffices preventing cld7 palmitoylation [33]. This model allowed deciphering palmitoylation-dependent activities of cld7 as well as differentiating between genuine versus driver activities of cld7. Both rescue lines confirmed a dominant role of palmitoylated cld7 in promoting motility and apoptosis resistance and pointed towards an involvement of cld7 in exosome biogenesis.

### Metastasis supporting activity of cld7 depends on palmitoylation

ASML^wt^ bearing rats die with lymph node and lung metastases after 5–6 wk. However, ASML-cld7^kd^ and -cld7^mPalm^ cells did not grow locally, did not reach the draining lymph node or the lung and 5/5 rats were tumor free after > 26 wk. Nonetheless, ASML-cld7^mPalm^ differ from ASML-cld7^kd^ cells starting to divide in soft agar at a comparable frequency to ASML^wt^ cells, but cells died after 1 wk. As the proliferation rate of ASML^wt^, -cld7^kd^ and -cld7^mPalm^ cells does not differ (data not shown), the finding points towards impaired apoptosis resistance of ASML-cld7^mPalm^.

Taken together, only palmitoylated cld7 supports tumor progression. Depending on the tumor type, the failure of non-palmitoylated cld7 to support metastatic growth could be due to integration in TJ. This could explain the discrepant findings on cld7 prohibiting [69, 70] vs. promoting metastasis [9, 10, 71–75].

### Cld7 palmitoylation, motility, invasiveness and the crosstalk with the cytoskeleton

Mass spectrometry of molecules co-immunoprecipitating with cld7 in ASML^wt^ cells confirmed the association with tetraspanins, CD44, the integrins α3(β1) and α6β4 and the association with cytoskeletal linker proteins [76]. The repeatedly described association with β1 [15, 77] is accompanied by a strong basolateral distribution, a reduction in pFAK and poor adhesion [77]. These reports are in line with our findings, which additionally point towards the requirement for cld7 palmitoylation to associate with integrins and downstream signaling cascades. Furthermore, cld7 amply associates with actin binding/organizing and actin polymerizing molecules. The associations with actin linker proteins mostly depend on cld7 palmitoylation, whereas the association with myosin and cytokeratins frequently is hampered by cld7 palmitoylation. This has consequences on cell motility, which is strongly reduced in ASML-cld7^kd^ and ASML-cld7^mPalm^ cells. We suggest the impact of palmitoylated cld7 on motility is supported by the association with tetraspanins and, possibly via Tspan8, with integrins [39–41].

Cld7 also associates with MMPs and uPAR [15, 78, 79], which contribute to motility by matrix protein degradation. Coimmunoprecipitation confirmed the association with uPAR and a weak association with MMP14. The uPAR association is exclusively observed with palmitoylation-competent cld7, i.e. depends on the GEM localization. Likely via the association with MMP14, MMP9 becomes recruited and activated, the gelatinolytic activity of ASML^wt^ cells significantly exceeding that of ASML-cld7^kd^ and ASML-cld7^mPalm^ cells. GEM-located palmitoylated cld7 also associates with CD147, which binds MMPs on neighboring cells [80] thereby promoting MMP activity. Corresponding changes being seen in ASML-Tspan8^kd^ and ASML-CD44v^kd^ cells [63, 64], we argue that the engagement of cld7 in invasiveness, similar to its contribution to motility, relies on associated GEM-located molecules.

Taken together, GEM-located palmitoylated cld7 supports motility and invasion by associating with integrins, tetraspanins, cytoskeletal linker proteins and proteases.

Tumor cells motility is frequently associated with EMT [81, 82]. Expression of several EMT-related genes is impaired in ASML-cld7^kd^ and -cld7^mPalm^ cells. Weak E-cadherin expression in ASML^wt^ cells is not affected. However, vimentin, FN and N-cadherin expression is reduced. Wnt1 is slightly downregulated in ASML-cld7^kd^ and, less pronounced, -cld7^mPalm^, but unaltered in -EpC^kd^ cells. In line with this finding is the downregulation of β-catenin and the pronounced β-catenin phosphorylation in ASML-cld7^kd^ and -cld7^mPalm^. Reduced recovery of nuclear β-catenin could account for impaired FN, Snail1, Snail2 and Twist expression [83]. However, only Snail1 recovery is impaired, whereas Slug expression is upregulated. Thus, in ASML cells Snail transcription [84] does not or not exclusively rely on β-catenin. Activation of EMT-related transcription factors can also proceed via integrins, ILK activation leading to NFkB nuclear translocation [86] or via activation of the src/p38MAPK pathway [87, 88]. uPAR, too, can induce EMT through activation of the PI3K/Akt pathway, src kinases, ERK/MAPK and myosin light chain kinase [89]. These alternative pathways of EMT transcription factor induction could be impaired in ASML-cld7^mPalm^ due to the reduced association with α3, α6β4 and uPAR. Finally, by not yet defined mechanisms, cld7 is engaged in Pten repression. High Pten expression in ASML-cld7^kd^ and -cld7^mPalm^ can account for downregulation of Snail, ZEB and Twist transcription [90].

Further explorations are required to precisely define the pathway(s), whereby palmitoylated cld7 supports transcription and/or nuclear translocation of EMT transcription factors, where the association with Notch could be the initial trigger. Nonetheless, the loss of EMT features of ASML-cld7^mPalm^ cells strongly argues for an engagement of palmitoylated cld7 in EMT.

### The engagement of cld7 in stress resistance

ASML cells are strikingly apoptosis resistant [62]. This relies on concerted activities of CD44v6 promoting activation of the MAPK and the PI3K/Akt pathway [63], on Tspan8 engaged in PI3K/Akt activation [64] and on cld7. There is no evidence for a direct engagement of cld7 in MAPK or JNK pathway activation. Instead, apoptosis resistance promoted by cld7 is accompanied by low level Pten expression [9]. Furthermore, there is evidence for palmitoylation-competent cld7 recruiting Pten repressing miRNA (unpublished finding). We now defined that only palmitoylated, GEM-located cld7 contributes to apoptosis resistance. Unexpectedly, apoptosis resistance was not rescued in ASML-cld7^mPalm^ cells, despite upregulated Pten phosphorylation. There are several possible explanations. First, Pten phosphorylation in ASML-cld7^mPalm^ cells is ineffective as Pten is not recruited into GEM. Mostly cytoplasmic cld7^mPalm^ associates with GSK3β and CK2, prominent Pten phosphorylating kinases [65]. Alternatively, not mutually exclusive, GSK3β and CK2 can also contribute to Pten stabilization [91, 92].

Taken together the contribution of cld7 to apoptosis resistance relies on Pten repression, supported by Pten inactivation. The inefficacy of cld7^mPalm^ to restore apoptosis resistance, despite pronounced Pten phosphorylation, may rely on the failure to recruit phosphorylated Pten towards GEM. Taking into account the multiple activities of Pten and the abundance of pathways regulating Pten transcription and activity [93] additional contributions of cld7 to Pten regulation cannot be excluded.

### Outlook: Cld7 and exosome biogenesis

Tetraspanins, located in so called tetraspanin-enriched microdomains, which are similar to GEM, play a major role in membrane invagination, the generation of early endosomes and their traffic through MVB towards the release as exosomes [67]. These findings fit to tetraspanins being constitutive exosome components [94]. We now described similar features for palmitoylated, GEM-located cld7. As cld7 and Tspan8 are loosely attached, are both located in GEM and are palmitoylated, we cannot sharply decipher, whether the two molecules work in concert or independently. Both cld7 and Tspan8 share the abundant association with chaperons [95], the association being mostly cld7 palmitoylation-independent. Furthermore, both - but Tspan8 more intensely - associate with integrins and membrane-integrated proteases [67, 73, 94]. Instead, cld7 has a strong affinity for proteasomal proteases, which might drive at least part of intraluminal vesicles into the degradation pathway rather than towards release. Tspan8 and cld7 abundantly associate with vesicle transporters of the rab family, some of these associations depending on cld7 palmitoylation, e.g. anti-rab5 and -rab7 coimmunoprecipitate cld7, but not cld7^mPalm^. On the other hand, Tspan8 and palmitoylation-competent cld7 do not or poorly associate with caveolin, which strengthens the assumption of a caveolin-independent internalization route. In concern about molecular complexes engaged in scission, fission and vesicle transport [96, 97], cld7 shares with tetraspanins [67] the association with clathrin, dynein and Snare proteins. Notably, too, colon cancer organoids deliver two types of exosomes. Only apical exosomes express tetraspanins, cld7 and EpC [22]. Coimmunoprecipitation of ASML^wt^ and -cld7^mPalm^ exosome lysates confirmed that the exosomal CIC markers EpC, CD44v6 and α6β4 are abundant in cld7 precipitates of ASML^wt^, but not -cld7^mPalm^ exosomes. Though it remains to be answered, whether palmitoylated cld7 actively contributes to exosome biogenesis, by its recruitment into exosomes the possibility should be taken into account that palmitoylated, GEM-located cld7 supports tumor progression also via exosomes.

**Figure 9:**
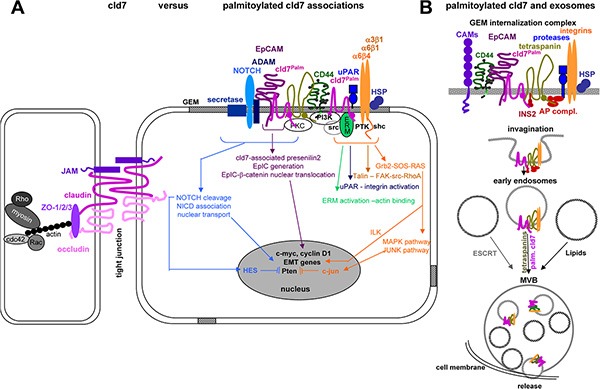
Overview of molecules associating and cooperating with palmitoylated cld7 (**A**) Only palmitoylated cld7 is enriched in GEM, where it associates in a direct protein-protein interaction with EpC. Interactions with additional transmembrane proteins, predominantly tetraspanins, integrins, CD44v6, transmembrane metalloproteases, ADAMs, uPAR and NOTCH likely are indirect and promoted by the special lipid composition of GEM and the catcher activity of tetraspanins, which may be first order partners for some of the listed membrane molecules. The majority of associations with cytoplamic molecules are indirect and promoted by the lipid composition of GEM. Thus, the multiple deficiencies associated with cld7^mPalm^ in migration, invasion, apoptosis resistance and EMT are due to exclusion from GEM (or recruitment into TJ) and persistence in the cytosol, the latter being demonstrated for Pten phosphorylation. (**B**) Cld7 is recovered in exosomes. There is evidence that only palmitoylated cld7 is recovered in those exosomes, where biogenesis/vesicle transport depends on tetraspanins rather than ESCRT complexes and lipids. Whether cld7 actively contributes to the biogenesis of these “tetraspanin-dependent” exosomes remains to be explored. Taken together, there is strong evidence that TJ-integrated cld7 and palmitoylated GEM-integrated cld7 fulfill non-overlapping activities. Whether cld7 palmitoylation is linked to the metastatic phenotype or is also observed in non-transformed cells and under which conditions remains to be explored.

As summarized in Figure 9, distinct to non-palmitoylated cld7, which is enriched in TJ complexes, palmitoylated cld7 is recruited into GEM, where it associates with EpC, tetraspanins, integrins, proteases, cytoskeletal components and signal transductions molecules. It is prone for internalization and release in exosomes. The multiple interactions of GEM-located protein complexes prohibit assigning activities as exclusively being palmitoylated cld7- versus GEM complexes-dependent. However, palmitoylated cld7 hardly shares activities with non-palmitoylated cld7 and only palmitoylated cld7 promotes metastasis.

## MATERIALS AND METHODS

### Cell lines

ASML [62], ASML-EpC^kd^, ASML-cld7^kd^ cells [9] were maintained in RPMI 1640/10% FCS w/wo 0.5 μg/ml G418. ASML-EpC^kd^ cells were transfected with pcDNΑ3.1(+)hygromycin plasmid containing EpC (ASML-EpC^resc^) or EpC mutated at G282 and A279 (ASML EpC^mAG^) or ASML-cld7^kd^ cells were transfected with cld7 mutated at AA184 and AA186 (palmitoylation site) using the pcDNΑ3.1(+)hygromycin plasmid (ASML- cld7^mPalm^). Where indicated, ASML-cld7^kd^ cells were transiently transfected with wt cld7 using the pcDNΑ3.1(+)hygromycin plasmid (ASML-cld7^resc^). Primers are listed in Supplementary Table S2. Stable rescue clones were established by single cell cloning and were cultured in RPMI 1640/10% FCS/0.5 μg/ml G418/120 μg/ml hygromycin.

### Antibodies and chemicals

See Supplementary Table S3.

### Exosome preparation

Cells were cultured (48 h) in serum-free medium. Cleared supernatants (2 × 10 min, 500 g, 1 × 20 min, 2000 g, 1 × 30 min, 10000 g) were centrifuged (90 min, 100000 g) and washed (PBS, 90 min, 100000 g). The resuspended pellet was purified by sucrose gradient centrifugation [67].

### Sucrose density gradient centrifugation

Cell lysates and exosomes in 2.5 M sucrose were overlaid by a continuous sucrose gradient (0.25 M–2 M) and centrifuged (15 h, 150000 g), collecting twelve 1 ml fractions.

### Palmitoylation assay

Palmitoylation of cld7 was determined using the IP-ABE method [98].

### IP, Western blot (WB)

Cells and exosomes were lysed for 30 min at 4°C with HEPES buffer, 1% Lubrol, 1 mM PMSF, 1 mM NaVO_4_, 10 mM NaF, protease inhibitor mix. During mild lysis with Lubrol protein complexes not relying on direct protein-protein interactions are not destroyed. Lysates were centrifuged (13000 g, 10 min, 4°C), mixed with antibody (1 h, 4°C) and incubated with ProteinG-Sepharose (1 h). Washed complexes/lysates, dissolved in Laemmli buffer, were resolved on 10%–12% SDS-PAGE. After protein transfer, blocking, blotting with antibodies, blots were developed with ECL.

### Protein identification

After SDS-PAGE, gels were stained with Coomassie-blue. Protein digestion, sample preparation, mass spectrometeric analysis by nanoLC-ESI-MS/MS on an LTQ orbitrap and database searches were performed as described [99].

Flow-cytometry followed routine procedures. Where indicated, cells or latex beads bound exosomes [67] were fixed and permeabilized. Samples were analyzed in a FACSCalibur using the CellQuest program.

### Zymography

Culture supernatant of ASML, -cld7^kd^ and -cld7^mPalm^ cells, starved for 24 h, was centrifuged (15 min, 15000 g). Aliquots of supernatant were incubated with Laemmli buffer (15 min, 37°C) and separated in a 10% acrylamide gel containing 1 mg/ml gelatin. After washing (2.5% Triton), gels were incubated in developing buffer (37°C, 48 h) and stained with Coomassie-blue.

### Confocal microscopy

Cells on glass-slides were fixed (4% paraformaldehyde, 20 min on ice), permeabilized (1% Triton-X100, 4 min, on ice), blocked (PBS/1% gelatin, 30 min, on ice), incubated with primary antibody (60 min, on ice), washed, incubated with fluorochrome-conjugated secondary antibody (60 min, on ice), blocked (IgG with irrelevant specificity of the same species as the primary antibody), incubated with a second, dye-labeled primary antibody and washed. Slides were mounted in Elvanol. Digitized images were generated using a Leica LMS780 microscope and the Carl Zeiss Vision software for evaluation. The Z-stack offers 30 positions through the depth of the cell. All pictures were taken at Z-stack 14–16. Depending on the quality of the antibody and the density of marker expression, the intensity for the green channel varied between 700–900 master gain values and for the red channel between 500–750 master gain values. The photosystem automatically generates the single fluorescence and overlay pictures. Only overlays are shown at a 50% reduction compared to the original size. Where indicated, selected fields were amplified 10-fold (5-fold compared to original). From the amplified field the membrane or the cytoplasm were encircled for evaluation of the Pearson correlation coefficient between the red and green channel using Image J (Rasband WS, ImageJ, US National Institute of Health, Bethesda, Maryland, USA http://imagej.nih.gov/ij/) [100].

### Histology

Snap frozen sections (5 μm) were fixed, incubated with antibodies, washed, exposed to biotinylated secondary antibodies and alkaline phosphatase conjugated avidin-biotin solution. Sections were counter-stained with H&E. Digitized images were generated using a Leica DMRBE microscope.

### Migration

Cells, in the upper part of a Boyden chamber (RPMI/0.1% BSA), were separated from the lower part (RPMI/20% FCS) by 8 μm pore size polycarbonate-membranes. After 16 h the lower membrane side was stained (crystal-violet), measuring OD595 after lysis. Migration is presented as % input cells. In an *in vitro* wound healing assay, a subconfluent monolayer was scratched with a pipette tip. Wound closure was controlled by light microscopy. For videomicroscopy, 5 × 10^4^ cells were seeded on laminin (LN)332-coated 24-well plates. Plates were placed under an Olympus IX81 inverse microscope with a Hg/Xe lamp, an incubation chamber (37°C, 5% CO_2_), a CCD camera (Hamamatsu) and a ScanR acquisition soft ware (Olympus, Hamburg, Germany). Two pictures (20-fold magnification)/chamber (2 ms exposure) were taken every 20 min for 12 h. Migration was quantified according to Manual_tracking plugin running in the open-source software Image J for 20 cells per well.

### Apoptosis

Cells (1 × 10^5^) were grown for 48 h in RPMI/10% FCS containing cisplatin. Survival was monitored by annexinV-APC/PI staining, MTT assay and ^3^H-thymidine uptake.

### Soft agar assay

Tumor cells in 0.3% agar were seeded on a preformed 1% agar layer counting colonies after 3 wk.

### *In vivo* assays

BDX rats received 1 × 10^6^ tumor cells intrafootpad (ifp). Rats were controlled weekly for local and draining lymph node tumor growth, short breathing or weight loss. Animals were sacrificed when draining nodes reached 2 cm diameter, rats lost > 10% weight or latest after 240 d. Animal experiments were Government-approved (Baden-Wuerttemberg, Germany).

### Statistics

*P* values < 0.05 (two-tailed Student's *t*-test, Kruskal-Wallis test) were considered significant.

## SUPPLEMENTARY MATERIALS FIGURES AND TABLES



## Abbreviations

ASML: BSp73ASML
CIC: cancer initiating cells
cld7: claudin-7
cld7^mPalm^: cld7 with a mutation in the palmitoylation site
EMT: epithelial-mesenchymal transition
EpC: EpCAM
EpC^mAG^: EpC with a AG point mutation
EpC^resc^: wt EpC rescue
ESI-MS/MS: electrospray ionization tandem mass spectrometry
FN: fibronectin
GEM: glycolipid-enriched membrane microdomains
ICD: intracellular domain
ifp: intrafootpad
IP: immunoprecipitation
kd: knockdown
LN: laminin
m: mutated
resc: rescue
TJ: tight junction
WB: Western blot
