# Supplementary material for: Palmitoylated claudin7 captured in glycolipid-enriched membrane microdomains promotes metastasis via associated transmembrane and cytosolic molecules
Source: Oncotarget. 2016 Apr 22;7(21):30659–77. doi: 10.18632/oncotarget.8928 (PMC5058708; doi:10.18632/oncotarget.8928)
Supplement: Supplementary file 1 [file oncotarget-07-30659-s001.pdf]

Palmitoylated claudin7 captured in glycolipid-enriched membrane microdomains promotes metastasis *via* associated transmembrane and cytosolic molecules

Supplementary Materials

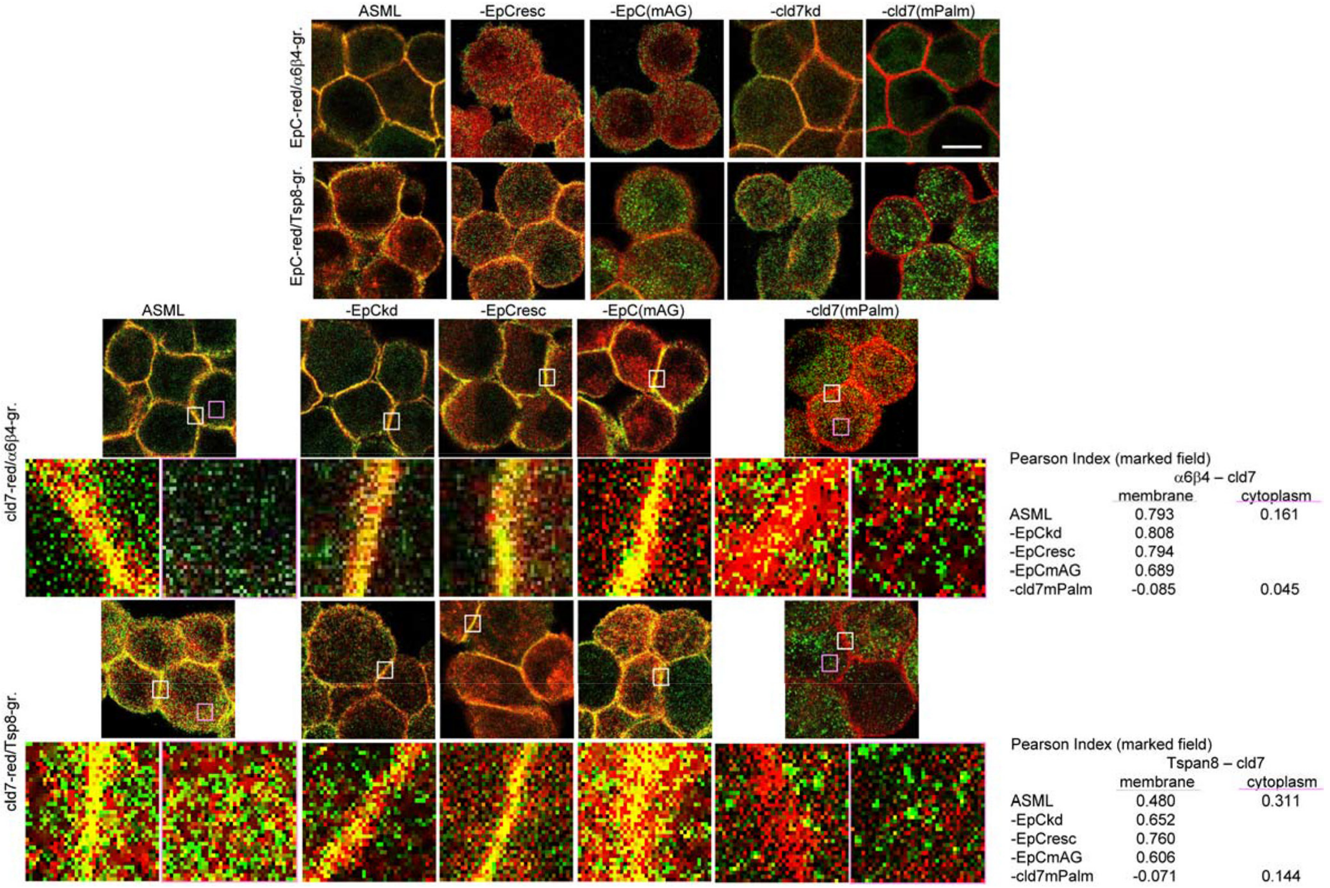

Supplementary Figure S1: The impact of cld7 on CIC marker and integrin expression in ASML cells: Colocalization of α6β4 (green) and Tspan8 (green) with cld7 (red) and EpC (red) was evaluated by confocal microscopy. Digital overlays are shown (scale bar: 10 µm). The indicated areas (membrane: white square, cytosol: pink square) were amplified 10-fold for better discrimination. The Pearson correlation coefficient is shown for the encircled membrane and cytosolic areas. Only palmitoylated cld7 strongly colocalizes with α6β4 and Tspan8.

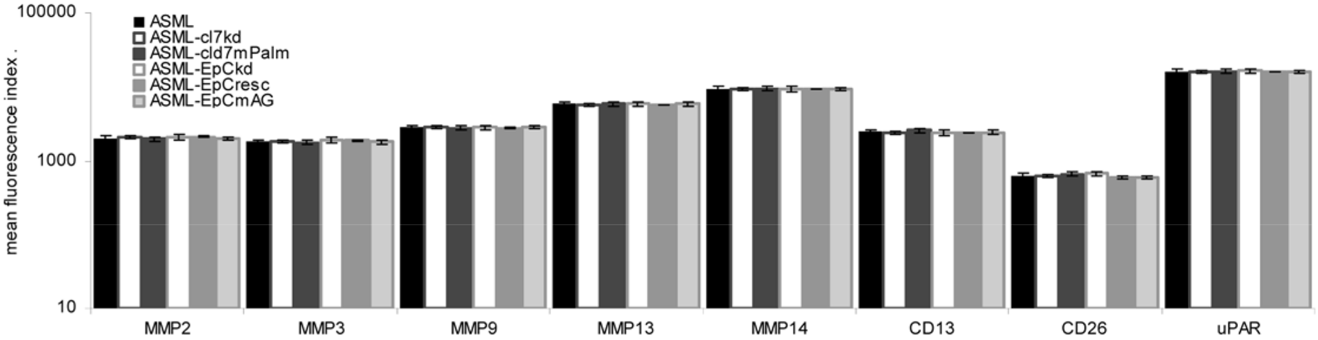

Supplementary Figure S2: The impact of cld7 on protease expression in ASML cells: Expression of the indicated proteases in wt, kd and rescue ASML cells was evaluated by flow cytometry. The mean fluorescence index (% stained cells × mean fluorescence intensity) ± SD is shown. A $cld7^{kd}$ or an EpC^{kd} does not influence protease expression.

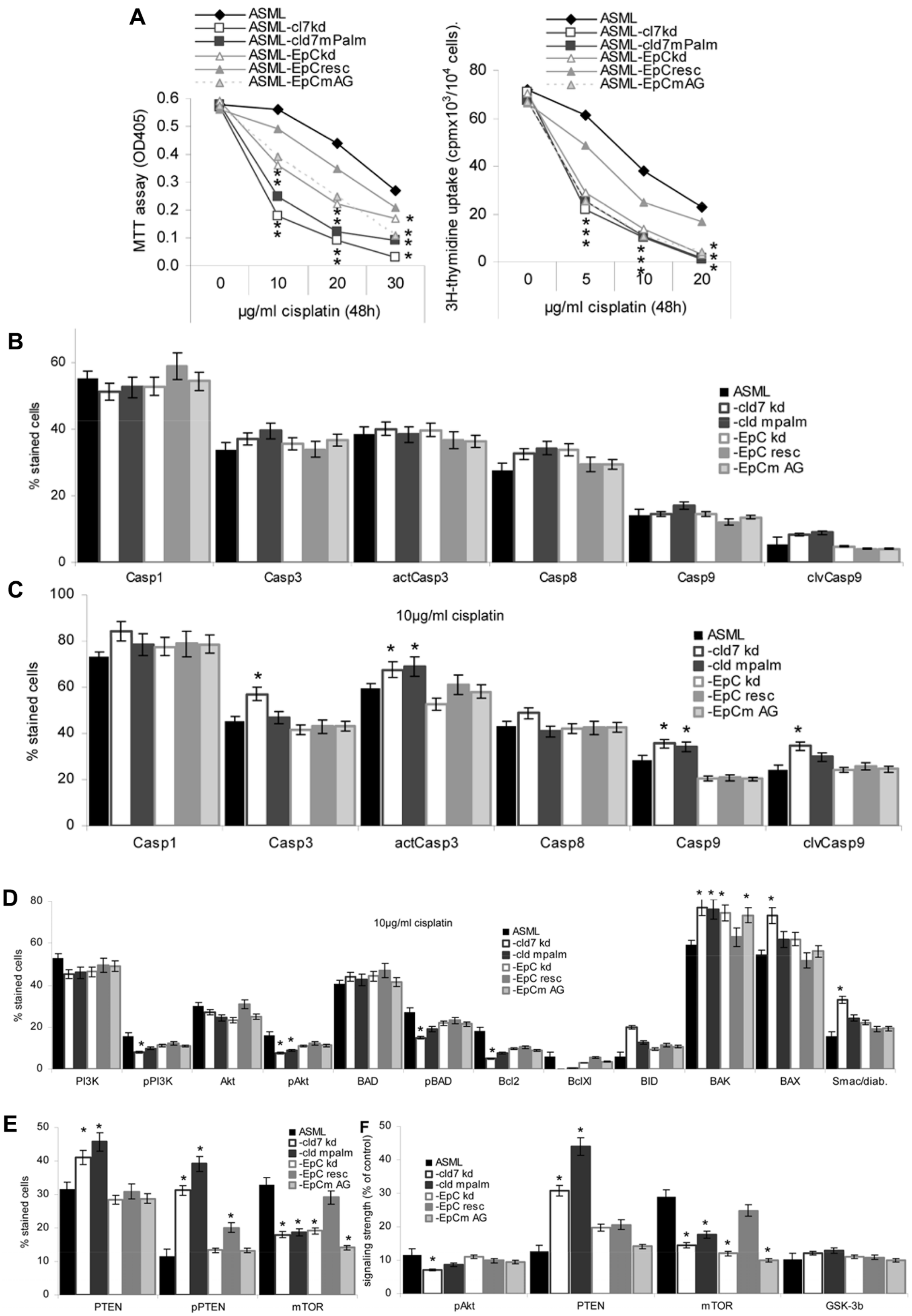

Supplementary Figure S3: Apoptosis effector and apoptosis-inducing molecule expression in dependence on cld7. (**A**) Wt, kd and rescue ASML cells were cultured in the presence of increasing amounts of cisplatin. Mitochondrial integrity (MTT assay, OD 405) and proliferative activity (^{3}H-thymidine uptake); Mean ± SD (triplicates) are shown, significant differences to wt cells: *(**B–F**) Untreated and cisplatin-treated (10 µg/ml) wt, kd and rescue ASML cells were stained with (B, C) caspase-specific, (D) PI3K/Akt pathway-specific and (E) Pten-, pPten, mTOR-specific antibodies. The mean % of stained cells ± SD (3 assays) is shown; (F) Array of apoptosis related signaling molecules, signal strength ± SD in comparison to the positive control is shown. (B–F) significant differences compared to wt ASML cells: *.

Most pronounced differences in ASML-cld7^{kd} and ASML-cld7^{mPalm} cells are seen in pAkt, BAK, BAX, Pten, pPten and mTOR expression in ASML-cld7^{kd} and -cld7^{mPalm} cells.

Supplementary Table S1A: Coimmunoprecipitation of cld7 with integrins, tetraspanins and components of the cytoskeleton

		wt		wt		EpCkd		EpCmAG		cld7mPalm		
		anti-EpC		anti-cld7		anti-cld7		anti-cld7		anti-cld7		
	Protein Description	signSequ.	% Cover	signSequ.	% Cover.	signSequ.	% Cover.	signSequ.	% Cover.	signSequ.	% Cover.	Function
Actn1	actinin, alpha 1	2	5.3	20	41.5	15	18.3	14	15.5	**2**	**2.4**	actin binding
Actn4	alpha-actinin-4			48	41.5	10	13.6	14	14.2	**0**	**0**	actin binding
Arpc1b	actin-related protein 2/3 subunit 1B	1	14.6	3	8.1	4	8.3	6	15.1	6	12.4	control of actin polymerization
Arpc2	actin-related protein 2/3 subunit 2							15	39	20	47	control of actin polymerization
Arpc3	actin-related protein 2/3 subunit 3							8	30.3	9	34.3	control of actin polymerization
Arpc5	Actin related protein 2/3 subunit 5			4	24.7	1	22.5	6	32.7	4	32.7	control of actin polymerization
Arpc1b	actin-related protein 2	2	5.8	4	8.1	3	12.4	13	22.6	10	19.3	control of actin polymerization
Cd151	CD151	1	9.9	0	0	0	0	0	0	0	0	migration, internalization
Cd44	CD44	11	8.5	8	10.5	13	14.5	8	9.1	13	13.7	adhesion, signaling
Cd81	CD81	1	8.5	0	0	0	0	0	0	0	0	migration, internalization
Cd82	CD82	1	4.1	0	0	0	0	0	0	0	0	migration, internalization
Cd9	CD9	16	15.5	2	15.5	2	15.5	2	15.5	2	15.5	migration, internalization
Cfl1	cofilin-1	3	33.7	3	31.2	14	57.2	7	30.1	20	57.2	actin modulator
Cldn1	claudin-1	2	7.1	1	7.1	1	7.1			2	7.1	cell-cell adhesion
Cldn4	claudin-4	2	13.3	2	13.3	1	13.3	2	5.7	2	13.3	cell-cell adhesion
Cldn7	claudin-7	8	17.3	8	17.3	8	17.3	10	17.3	12	17.3	cell-cell adhesion, signaling
Coro1c	coronin-1C	0	0	12	23.6	3	15	10	21.5	25	38.8	actin-binding kinesin complex
Dsp	desmoplakin	9	5.1					9	4.3	7	3.1	anchors filaments to desmos.
Dstn	destrin	2	14.5	2	14.5	2	14.5	2	14.5	2	14.5	actin depolymerizing factor
Emd	emerin	6	20.8	2	19.2	2	5.8	6	16.9	0	0	cytosk. membrane anchoring
Ezr	EZRIN	0	0	15	21.5	11	14.2	7	21.5	17	16.4	cytoskeletal linker protein
Flna	filamin, alpha	22	18	27	21.6	22	18	27	21.6	27	21.6	crosslinks actin filaments
Flii	flightless-1 homolog	1	0.9	2	3.4	7	4	5	6.1	9	14	actin binding

Gsn	gelsolin	7	14.2	76	36	29	33.5	85	36.4	90	43.2	disassembly of actin filaments
Itga6	integrin alpha 6	3	3	2	5.4	0	0	0	0	0	0	matrix/cell adhesion, signaling
Itga3	integrin alpha-3	11	8	6	4.4	1	2.1	5	3.3	5	3.3	matrix/cell adhesion, signaling
Itgb4	integrin beta-4	14	26.3	5	14.4	1	4.5	3	7.8	4	5.5	matrix/cell adhesion, signaling
Krt5	keratin, type I cytoskeletal 5	10	16.5	4	9.9	10	16.5	4	9.9	4	9.9	cytoskeleton organization
Krt8	cytokeratin 8 polypeptide	2	3.8	2	7.2	2	3.8	2	7.2	2	7.2	cytoskeleton organization
Krt10	keratin, type I cytoskeletal 10	36	17.9	8	14.6	35	16.2	89	20.2	**129**	**19.8**	cytoskeleton organization
Krt14	keratin, type I cytoskeletal 14	12	19.8	6	5.4	8	13.2	22	13.8	41	18.8	cytoskeleton organization
Krt16	keratin, type I cytoskeletal 16	4	10.2	3	7.8	6	14.3	20	13.6	33	16.8	cytoskeleton organization
Krt19	keratin, type I cytoskeletal 19	20	23.8	7	9.9	11	11.2	23	18.4	31	21.4	cytoskeleton organization
Krt42	keratin, type I cytoskeletal 42	8	19.5					19	14.2	33	14.8	cytoskeleton organization
Krt1	Keratin, type II cytoskeletal 1	37	8.6	21	7.4	45	8.5	61	8.6	89	9.1	cytoskeleton organization
Krt20	keratin, type II cytoskeletal 2	12	4.7	2	2	13	4.8	33	5	39	6.3	cytoskeleton organization
Krt5	keratin, type II cytoskeletal 5	22	13.7			15	12.2	48	16	59	20	cytoskeleton organization
Krt6	keratin, type II cytoskeletal 6A	28	7.1	16	5.8	27	7.6	58	9.6	72	22.6	cytoskeleton organization
Krt75	keratin, type II cytoskeletal 75							36	7.3	50	9.4	cytoskeleton organization
Kb15	keratin, type II cytoskeletal					20	6	37	6	57	8.3	cytoskeleton organization
Capg	macrophage-capping protein	1	3.7	6	19.5	6	19.5	2	7.7	12	29.9	barbed-end actin filament
Lad1	ladinin1									7	20.8	filament anchoring
Msn	moesin	4	7.3	9	21.5	4	7.3	8	10.1	**0**	**0**	cytoskeletal linker protein
Myh10	myosin, heavy polypeptide 10	322	54	466	60	301	56	392	62	697	63	actin organization
Myh11	myosin-11			147	12	43	53			174	14	actin organization
Myh14	myosin-14	231	45	361	45	212	50	454	61	701	62	actin organization
Myh9	myosin-9	659	61	883	61	341	56	656	65	1148	67	actin binding
Myl2	myosin light chain 2									114	63	actin organization
Myl3	myosin light chain 3	13	12	17	29	3	12	28	34	30	41	actin organization
Myl6l	myosin light polypeptide 6									163	76	actin organization
Myo1b	myosin I heavy chain	31	18	41	23	4	6.9	47	19	75	39	actin organization

Myo1d	unconventional myosin-Id					3	8.1	32	21	42	29	actin binding
Myo6	Myosin VI	5	7.1	2	5	2	7.2	11	11	5	6.4	actin movement
Pkp1	plakophilin-1	5	8.8	0	0	0	0	0	0	0	0	desmosomes
Pfn1	profilin	2	27.9	2	20	17	57.9	26	57.9	30	57.9	small actin binding protein
Rhoa	ras homolog family member A	2	18.7	2	9.8	2	18.7	1	9.8	**0**	**0**	actin cytosk. organization
S100a11	S100-A11	3	37.8	1	26.5	1	16.3	1	26.5	1	16.3	motil., invas., tubulin polym.
S100a10	S100-A10	4	35.8					2	17.9	5	21.1	motil., invas., tubulin polym.
S100a11	S100-A11	7	37.8	2	26.5	1	16.3	6	27.6	5	16.3	motil., invas., tubulin polym.
S100a6	S100-A6	2	30.3	1	30.3	0	2.8	0	2.8	1	6.9	motil., invas., tubulin polym.
Testin	testin	60	55.4	74	62.5	52	54.2	92	65.2	66	55.8	component junct. complexes
Tspan8	tetraspanin-8	18	30.2	5	16.6	9	24.3	10	15.7	10	15.7	internal., migrat., signaling
Tmod3	tropomodulin-3	7	28.4	9	41.2	7	28.4	9	41.2	9	41.2	actin binding
Tpm3	Tropomyosin alpha-3	7	35.1	59	59.3	13	31	68	52	73	55.2	actin-binding protein
Tpm4	tropomyosin alpha-4	4	30.6	37	51.2	9	23.8	57	67.3	61	55.6	actin-binding protein
Tuba1a	tubulin alpha	42	30.7	44	26.7	40	40.8	60	47.7	68	45.7	cytoskeleton
Tubb6	tubulin beta-6 chain	0	0	32	22.1	32	29.1	51	32	51	35.1	cytoskeleton

Supplementary Table S1B: Coimmunoprecipitation of signaling molecules with cld7

		ASML		-cld7kd		-EpC (mAG)		-cld7 (mPalm)		ASML		-EpCkd		-EpC (mAG)		-cld7 (mPalm)		
		anti-EpC IP[a]								anti-cld7 IP[a]								
ID	function	sign.M	% Cov	sign.M	% Cov	sign.M	% Cov	sign.M	% Cov	sign.M	% Cov	sign.M	% Cov	sign.M	% Cov	sign.M	% Cov	Protein Description
ANP32A	signal., phosphatase inhib.	4	14	1	7	3	13	1	5	0	14	2	18	2	5	3	11	acid.leucine-rich phosphopr.32A
C3	signal transduction	6	3	7	4	6	3	7	3					8	3	6	4	Complement C3
Cd59	signal transduction	7	23	7	23	3	14	5	23	5	23	7	23	10	23	10	23	CD59
Clic1	signal., transfer., reduct.	4	17	3	22	1	8			1	8	2	12	6	24	6	30	chloride intracell. channel protein 1
Csnk2a1	signaling (Wnt)	3	13	19	53	20	40	15	48					2	2	2	6	casein kinase II subunit alpha
Dapk3	ser/thr kinase, apoptosis	2	3	3	6	7	12	13	27									Death associated protein 3
Cdk1	serine/ threonine kinase									4	11			8	19	6	10	cyclin-dependent kinase 1
Far1	seri/thr kinase inhibitor													5	9	9	13	fatty acyl-CoA reductase 1
Gnb1	signaling (PI3K)	23	30			6	14	9	23	8	25	4	17	11	16	18	32	G protein beta polypeptide 1
Gstp1	signal. (neg. JUN,MAPK)	22	39	23	52	16	34	16	43	33	39	49	58	27	39	34	43	glutathione S-transferase P
Hmgb2	signal transduction	6	25	2	17					12	32	3	22			5	22	high mobility group protein B2
Khdrbs1	signal transduction			6	8	8	7	13	8					6	5	5	3	signal transd.-assoc. protein 1
Lgals3	signal transduction	6	17	6	31	6	26	1	18	4	17	4	16	2	17	5	41	galactoside-binding lectin 3
Met	signal transduction											5	6	4	2	2	1	c-met

Myh7b	signaling (Wnt)									12	2			6	2	11	4	myosin, heavy polypeptide 7B
Pdia3	signal., oxidase, isomerase	8	14					0	4	12	19	12	17	8	13	23	31	disulfide-isomerase A3
Pdia4	signal., oxidase, isomerase									6	12	7	21	2	3	0	0	disulfide-isomerase A4
Prkcz	signaling (Wnt)	7	34	17	36	18	38	8	35	6	33	12	34	20	42	15	43	protein kinase C zeta
Rap1b	signal transduction	3	19	4	13	2	13	3	13	3	13	8	21	2	20	6	34	Rap1b
Ras	signal transduction	15	22	21	45											21	53	rat sarcoma virus oncogene
Rras	signal transduction	3	13	7	18			3	16	5	20			3	11	8	30	R-Ras
S100a4	signal transduction	2	9	3	17	3	26	4	35					4	26	2	26	S100-A4
Siah1	signal. (Wnt), ubiqu. ligase			6	13	17	24	26	36			2	7					siah E3 ubiquitin protein ligase 1
Sub1	signaling (SMAD)	11	70	11	42	13	43	6	33	12	46			2	17	0	0	SUB1 oncodevelopmental protein
Tfg	signal transduction	6	17	32	45	32	45	22	39	7	17	14	39	60	46	26	39	Trk-fused gene
Ube2n	signal., ubiquitination, lysis	2	7	4	24	5	37			3	14	2	37	2	14	6	36	ubiquitin-conjug. enzyme E2 N

Supplementary Table S1C: Cld7 and EpC associated transporter and carrier proteins

			ASML		-cld7kd		-EpC(mAG)		-cld7(mPalm)		ASML		-EpCkd		-EpC(mAG)		-cld7(mPalm)	
			EpC IP[a]		EpC IP[a]		EpC IP[a]		EpC IP[a]		cld7 IP[a]		cld7 IP[a]		cld7 IP[a]		cld7 IP[a]	
ID	Protein Description	funct	sign.M	% Cov	sign.M	% Cov	sign.M	% Cov	sign.M	% Cov	sign.M	% Cov	sign.M	% Cov	sign.M	% Cov.	sign.M	% Cov
Akr1b1	aldose reductase	trp	10	7	12	16			4	11	8	12	7	21	3	9	11	13
Akr1b8	aldose reductase-related protein 1	trp	4	10	13	40			2	23	5	15	3	19	3	10	13	34
Aqp1	aquaporin 1	trp	8	15							9	11	6	11			2	3
Atp1a1	Na,K-ATPase subunit alpha-1	trp	7	8	2	2	4	6	3	5	9	13					15	18
Atp2a2	endoplasmic reticulum Ca2+-ATPase	trp									2	3	4	8	1	2	3	5
Atp5a1	ATP synthase subunit a	trp	15	27	24	37	17	24	17	29	11	24	14	24	16	25	18	25
Atp5e	ATP synthase subunit e	trp	5	42	8	51	8	52	7	51					2	35	3	32
Atp5o	ATP synthase subunit o	trp			5	10	4	5	4	10							5	21
Bcap29	B-cell receptor-associated protein 29	trp	7	25	16	33	15	31	5	18	6	20	3	17	9	25	2	10
Bcap31	B-cell receptor-associated protein 31	trp	8	23	15	23	21	44	9	16	5	23	10	23	13	22	9	18
Cabp1	neg.regul.Ca^{++} channels	trp	10	19	8	26	12	25	3	12	8	17	10	20	14	34	10	21
Clic1	chloride intracellular channel protein 1	trp	4	17	3	22	1	8			1	8	2	12	6	24	6	30
Kpnb1	importin subunit beta-1	trp											5	9	5	6	2	1
Praf2	PRA1 family protein 2	trp	2	6	3	12	4	16	1	6	2	6	2	6	6	12	2	6
Reep6	receptor expression-enhancing protein 6	trp	2	6	5	18	7	22	2	9	1	6	6	22	4	11	6	15
Slc16a1	monocarboxylate transporter 1	trp	3	5	3	5	3	5	3	5	4	5	2	3	4	5	4	5
Slc22a1	solute carrier family 2, glucose transp. 1	trp									2	6	3	3	5	4	0	0
Slc25a13	solute carrier family 25, member 13	trp	1	2							2	3						
Slc25a3	Solute carrier family 25 member 3	trp	4	12							2	15						
Slc25a4	solute carrier family 25, member 4	trp	6	22	8	22	10	23	3	11	5	25	3	23	3	10	8	14
Slc25a5	solute carrier family 25, member 5	trp	8	15	11	22	8	31	6	14	10	22	3	27	4	14	10	18

Slc3a2	4F2 cell-surface antigen heavy chain 1	trp	3	5							2	2						
Tm9sf2	transmembrane 9 superfamily member 2	trp	3	5					9	23	3	7					6	18
Tnpo1	transportin1	trp															23	57
Vdac1	voltage dependent anion channel	trp									5	19	2	23			2	9
Acbd4	acyl-CoA-binding protein	carr			2	32	3	32	5	32			1	32	1	32	2	32
Afp	Alpha-fetoprotein	carr	4	1							5	1						
Alb	serum albumin	carr	76	7	65	7	42	6	39	7	19	7	37	11	42	10	35	8
Mfge8	lactadherin isoform 2	carr			1	6	7	8							1	2	3	15
Mfi2	melanotransferrin	carr			2	2	1	2			2	8	3	8	4	4	0	0
Tmed2	transmembrane emp24 domain 2	carr	3	16	7	15			5	31	2	10	5	18	6	22	2	9
Tmed4	transmembrane emp24 domain 4	carr	8	19	8	19	8	19	7	19	6	16	3	16	4	19	10	16
Tmed9	transmembrane emp24 domain 9	carr	8	18	11	31	12	31	11	23	5	16	5	16	6	23		
Uba1	ubiquitin-like modifier-activating enzyme 1	carr	3	3							10	8	9	12				

Supplementary Table S1D: Coimmunoprecipitation of chaperones with cld7

		ASML		-cld7kd		-EpC(mAG)		-cld7(mPalm)		ASML		-EpCkd		-EpC(mAG)		-cld7(mPalm)	
		anti-EpC IP[a]								anti-cld7 IP[a]							
ID	Protein Description	sign.M	% Cov		% Cov	sign.M	% Cov	sign.M	% Cov	sign.M	% Cov	sign.M	% Cov	sign.M	% Cov	sign.M	% Cov
Canx	calnexin	2	4	0	8	7	9			2	9	8	18	3	7	5	9
Cct2	chaperonin containing TCP1 beta	6	10	6	12	1	5			7	9	13	27	13	18	15	23
Cct3	chaperonin containing TCP1 gamma	2	5	4	4	3	6			3	6	6	13	4	4	8	8
Cct4	chaperonin containing TCP1, delta									9	10	10	21	9	13	16	17
Cct5	chaperonin containing TCP1, epsilon	3	6							6	8	6	15	3	7	11	18
Cct6a	chaperonin containing TCP1, zeta 1	2	7	2	12	4	11			4	12	5	16	11	15	13	18
Cct8	chaperonin containing Tcp1, subunit 8	3	10	3	4	4	7	1	9	6	9	12	14	13	23	0	0
Dnajp9	dnaJ homolog subfamily C member 9	23	31	2	4	3	8	2	8	22	39	1	4	8	21	2	8
Dnajpb11	dnaJ homolog subfamily B11			2	10	1	3			2	5			2	3		
Dnajpb6	dnaJ homolog subfamily B member 6			4	7							1	3	5	9	4	7
Fkbp4	FK506 binding protein 4	3	8	2	19	1	19			6	10	2	11	1	11	0	0
Hsp90aa1	heat shock protein 90, alpha	68	29	61	35	52	25	42	22	86	34	97	42	74	39	91	40
Hsp90b1	endoplasmin precursor	15	6	26	8	16	6	9	6	19	12	34	27	26	24	18	14
Hspa13	heat shock protein 70, member 13									47	42	86	51	145	54	97	56
Hspa1a	heat shock protein 70	26	28	54	47	29	29	30	26	18	11	14	8	12	11	11	11
Hspa1b	heat shock protein 70kd 1B	73	43			57	41			101	46	84	46	79	43	0	0
Hspa4	heat shock 70 kDa protein 4									5	7	2	7	7	7	7	7
Hspa5	78 kDa glucose-regulated protein	22	34	27	35	27	36	20	26	37	35	47	46	60	47	53	43
Hspa9	heat shock protein 90, alpha	6	4							11	6	7	3	4	6	0	0
Hspd1	heat shock 60kDa protein 1	24	30							39	32	59	52	33	40	74	46
Hspe1	chaperonin 10	7	81	12	54	5	44	8	44	4	52	3	54	11	16	8	54
Hyou1	Hypoxia up-regulated protein 1	1	3							2	2	4	7	4	5	0	0
LOC100912256	hsc70-interacting protein	2	3							2	6	3	11	2	3	4	15
Npm1	nucleophosmin	9	33	6	19	13	33	5	16								
Set	SET nuclear proto-oncogene	25	23	10	17	9	17	12	17	2	10	7	19	5	13	9	13
Stip1	stress-induced-phosphoprotein 1	1	4	1	5					5	10	9	21	8	17	2	5
Tcp1	T-complex protein 1 subunit alpha	3	7	3	8	2	6	1	8	9	12	15	27	3	10	22	33
Ywhag	14-3-3 protein gamma	7	25	14	33	14	35	12	35	5	22	6	16	9	24	10	25

Supplementary Table S1E: Coimmunoprecipitation of vesicle transporters with cld7

		ASML		-cld7kd		-EpC(mAG)		-cld7(mPalm)		ASML		-EpCkd		-EpC(mAG)		-cld7(mPalm)	
		anti-EpC IP								anti-cld7 IP							
**ID**	**Protein Description**	**sign.M**	**% Cov**	**sign.M**	**% Cov**	**sign.M**	**% Cov**	**sign.M**	**% Cov**	**sign.M**	**% Cov**	**sign.M**	**% Cov**	**sign.M**	**% Cov**	**sign.M**	**% Cov**
Cav1	caveolin 1			6	30	2	22	5	29			0	24	5	30	13	45
Cav2	caveolin 2	2	7	1	19	4	44	1	11	2	7	2	23	3	7	8	55
Erp29	endoplasmic reticulum res. protein 29	6	16	4	8	4	8	3	8	4	20	4	20	8	20	3	14
Lamp2	lysosome membrane protein 2									**1**	**3**	**2**	**2**	**1**	**2**		
Lman2	lectin, mannose-binding2			5	11	1	11	3	15			2	12	10	24	4	20
Myof	myoferlin	22	13	4	3	16	7	2	1	15	8	12	6	31	15	3	1
Nipsnap1	nipsnap1	2	8	4	10	3	10	4	10	2	8	1	3	3	11	2	5
Rab1b	Rab-1B			21	46	14	27	10	15	11	28	11	42	21	62	26	12
Rab2a	Rab-2A	9	31	9	41	6	24	5	18	9	26	12	32	11	44	8	36
Rab5b	Rab-5B			4	12	3	12	3	12			3	32	8	27	4	17
Rab5c	Rab-5C	6	35	8	32	7	26	6	26	6	24	6	31	8	24	5	16
Rab7a	Rab-7a	6	29	12	50	9	50	5	24	6	33	5	42	23	57	6	36
Rab14	Rab-14	9	17	12	27	9	20	7	12	5	24	8	31	13	32	11	32
Rab18	Rab-18	2	7	12	44	6	24	5	18	1	13	3	24	14	43	6	18
Rab25	RAB-25									**2**	**20**	**0**	**10**	**10**	**28**	0	0
Rtn4	reticulon 4	2	5	2	4	2	4			1	3	2	5	2	8	0	0
Sdcbp	syntenin-1													2	10	2	4
Sec13	transport protein SEC13	2	7	4	3			2	3					3	3	4	3
Sec31a	transport protein Sec31A	3	1	3	1	7	1	7	2	2	1	2	1	2	1	0	0
Srp19	signal recognition particle 19			4	23	7	39	6	33								
Syngr2	synaptogyrin-2			2	4	2	4	2	4					2	4	2	4
Tmed5	transmembrane emp24 domain 5	1	7	3	7	2	5	2	3					1	3	5	10
Tmed7	transmembrane emp24 domain 7	4	13	5	20	3	16	6	20	1	10	3	10				
Vapa	VAMP-associated protein A	0	10	6	27	5	18	11	23					1	10	7	22
Vapb	vesicle-associated membrane protein B			4	11	3	11	3	11					2	19	5	11

Supplementary Table S1F: Coimmunoprecipitation with transporter complexes (exosome components)

		ASML		-cld7kd		-EpC(mAG)		-cld7(mPalm)		ASML		-EpCkd		-EpC(mAG)		-cld7(mPalm)		
		anti-EpC IP								anti-cld7 IP								
ID	Protein Description	sign.M	% Cov	sign.M	% Cov	sign.M	% Cov	sign.M	% Cov	sign.M	% Cov	sign.M	% Cov	sign.M	% Cov	sign.M	% Cov	function
Atl3	atlastin-3			3	12	0	3					5	10	1	5			dynamin like GTPase
Bet1	BET1 homolog	1	15	5	25			4	25	1	15			1	15	3	25	vesicles docking
Cltc	clathrin heavy chain 1	36	21	28	21	40	23	5	5	18	11	80	37	19	14	65	32	vesicle coat, intracell. traffic
Copa	coatomer complex alpha	132	43	170	60	160	58	120	47	83	33	168	56	146	46	127	51	vesicular coat
Copb1	coatomercomplex beta 2	35	33	68	47	46	50	45	38	36	32	72	46	51	39	64	51	vesicular coat
Cope	coatomer complex epsilon	27	67	41	77	47	55	38	84	11	37	22	35	27	48	37	56	vesicular coat
Dync1h1	dynein 1 heavy 1	12	3			1	1			17	4	16	5	14	5	20	4	ATPase, organelle movem.
Dync1i2	dynein interm. 2B											3	6	2	3	3	6	vesicle movement
Dync1li2	dynein 1 light interm. 2													3	6	3	4	vesicle movement
Gna11	guanine nucl.-bind.α11	2	11	6	15			4	7					1	7	2	5	transmembr signalling
Gnai2	guanine nucl.-bind.α2	14	34	12	25	11	21	9	18	25	34	15	25	19	32	26	31	adenylate cyclase regulat.
Gnai3	guanine nucl.-b.G(k)α	6	12	10	18	9	13	9	21	14	20	11	20	9	23	29	33	transmembr signalling
Gnb1	G protein β1	23	30			6	14	9	23	8	25	4	17	11	16	18	32	transmembr signalling
Jup	junction plakoglobin	6	15					36	28									complex with cadherins
Tuba1c	tubulin α1C chain	42	31			48	53			44	27	40	41	60	48	68	46	intracellular transport
Tubb6	tubulin β6 chain									32	22	32	29	51	32	51	35	intracellular transport
Vamp2	vesicle ass. membr.prot.2B	3	13	6	18					1	13			3	24	0	0	vesicle fusion & exocytosis
Vamp5	vesicle-ass. membr.prot.5	2	13			2	13			1	13			2	13	2	13	vesicle traffic
Vamp8	vesicle-ass. membr.prot.8	2	22	1	24	7	24	2	24	0	14			4	24	2	24	vesicle fusion
Ykt6	prenyl. SNARE Ykt6p	6	15	8	24	1	7	2	7	3	15			4	18	8	24	vesicle transport

Supplementary Table S1G: Coimmuoprecipitation with intracellular proteases

		ASML		-cld7kd		-EpC(mAG)		-cld7(mPalm)		ASML		-EpCkd		-EpC(mAG)		-cld7(mPalm)		
		anti-EpC IP								anti-cld7 IP								
ID	Protein Description	sign.M	% Cov	sign.M	% Cov	sign.M	% Cov	sign.M	% Cov	sign.M	% Cov	sign.M	% Cov	sign.M	% Cov	sign.M	% Cov	
Pebp1	PE binding protein									3	28	7	25	4	28	3	14	endopep. inhib.
Ctsd	Cathepsin D	4	5							3	20	4	18	3	13	13	15	protease
Ctsz	Cathepsin Z	1	9							2	12	2	9	2	3	2	12	protease
Lonp1	lon protease homolog	1	1	4	6					6	6	3	5			0	0	protease
Pa2g4	prolif.-associated 2G4			8	14	30	42	32	36	2	3			1	3	2	3	protease
Park7	parkinson protein 7											3	33	1	15	3	46	protease
Pdcd6	programmed cell death 6	4	25	9	31	5	22	6	33	6	41							protease
Pdia3	disulfide-isomerase A3	8	14							23	31	12	19	12	17	8	13	protease
Pdia4	disulfide-isomerase A4	1	2							6	12	7	21	2	3	0	0	protease
Prss1	anionic trypsin-1 precursor			16	8	8	8	7	8			91	61	11	8	15	8	protease
Sec11a	sec11A, catalytic subunit	3	10	7	24	7	20	6	16	2	5	6	20	2	5	3	13	protease
Sp120	serine protease 120	35	19	29	12	32	17	36	29	12	13	12	12			10	12	protease
Sri	sorcin	1	12	8	25	2	15	3	11					4	21	2	10	protease
Testin	testin	60	55	28	48	65	55	26	52	74	63	52	54	92	65	66	56	protease
Psma1	proteasome alpha type-1	2	6	4	20					2	6	2	15	4	10	5	24	protease
Psma2	proteasome alpha type-2	1	9	3	23					3	16	1	6	3	11	1	6	protease
Psma3	proteasome alpha type-3	2	9	4	11	2	4	1	4	2	5	3	13	3	10	4	11	protease
Psma4	proteasome alpha type-4	5	17	2	7	2	4	2	4	2	17	2	10	3	14	4	10	protease
Psma6	proteasome alpha type-6	5	19	6	20	2	13	3	9	4	33	6	29	9	27	5	29	protease
Psma7	proteasome alpha type-7	6	16	8	30	5	16	3	12	5	22	7	30	10	36	0	0	protease
Psmb1	proteasome beta type-1	3	14	6	24	2	8			3	14	6	24	6	24	0	0	protease
Psmb2	proteasome beta type-2	2	8							2	8	2	11	2	16	4	21	protease
Psmb3	proteasome beta type-3	5	18	4	11	4	11	1	3	5	18	1	15	5	11	0	0	protease
Psmb4	Proteasome beta type-4	3	10							6	21	3	21	1	7	3	20	protease
Psmb8	proteasome beta type-8											4	13			4	20	protease
Rpn1	ribophorin I			7	14	14	26					11	22	12	20	0	0	protease compl.

[a] The number of specific peptide hits and the % of the protein sequence covered by identified peptides is shown. Pronounced association with EpC: blue, pronounced association with cld7: red; lost or strongly reduced association with palmitoylation-deficient cld7: violet, pronounced association with palmitoylation deficient cld7: khaki.

Supplementary Table S2: Primers

EpC and EpC^{mAG} rescue:	
AflIIIPstImut for:	CTGCCCTGCAGGACACGTTCGCATCTCGATACATGC
AflIIIPstImut rev:	GCATGTATCGAGATGCGAACGTGTCCTGCAGGGCAG
Cld7 and cld7ΔPalm184 rescue:	
for:	GGAGGGGCCCTGCTCTCTTTCCTCCTCCCCCGGCA GTGAAAGCAAAG
rev:	CTTTGCTTTCACTGCCGGGGGAGGAGGAAGAGAG CAGGGCCCCTCC

Supplementary Table S3: Chemicals and antibodies
S3A Chemicals

Substance	Dose	Supplier
AnnexinV-FITC/-APC	variable	Becton Dickinson, HD, G
Cisplatin	1–30 µg/ml	Sigma, Munich, G
Matrigel	invasion: 1:5	Becton Dickinson, HD, G
Phalloidin	0.5 µg/ml	Becton Dickinson, HD, G
PI	variable	Becton Dickinson, HD, G
PMA	10^{-8} M	Sigma Munich, G

S3B Antibodies

Antibody	Origin	Supplier
Actin	mouse	Becton Dickinson, HD, G
Akt	mouse	Becton Dickinson, HD, G
Annexin II	mouse	Becton Dickinson, HD, G
BAD	mouse	Becton Dickinson, HD, G
BAK	mouse	Biozol, Eching, G.
BAX	mouse	Becton Dickinson, HD, G
β-catenin	rabbit	Becton Dickinson, HD, G
Bcl2	mouse	Becton Dickinson, HD, G
BclXl	mouse	Becton Dickinson, HD, G
BID	mouse	Becton Dickinson, HD, G
Cathepsin D	rabbit	Santa Cruz, HD, G
casein kinaseβ	mouse	Becton Dickinson, HD, G
Caspase1	rabbit	Santa Cruz, HD, G
Caspase3	mouse	Becton Dickinson, HD, G
Caspase3 activated	rabbit	Becton Dickinson, HD, G
Caspase8	rabbit	Becton Dickinson, HD, G
Caspase9	mouse	Becton Dickinson, HD, G
Casp.9 cleaved	rabbit	Cell Signalling, Frankfurt, Germany

caveolin	rabbit	Becton Dickinson, HD, G
CD11b(αM)	mouse	clone Ox42 (EAACC)[a]
CD13	mouse	Becton Dickinson, HD, G
CD26	mouse	ImmunoTools, Friesoythe, G
CD44v6 (A2.6)	mouse	ref [1]
CD49c (α3)	mouse	clone Ralph3.1 (EAACC), BD
CD49f/CD104 (B5.5)	mouse	ref [1]
CD87	mouse	Calbiochem, Darmstadt, G
CD104	rabbit	Becton Dickinson, HD, G
CD147	mouse	Becton Dickinson, HD, G
CD151	rabbit	ref [2]
Ck2	rabbit	Stressgen, Canada
clathrin	mouse	Calbiochem, Darmstadt, G
cld7	guinea pig	ref [3]
Dynamin	rabbit	Santa Cruz, HD, G
E-cadherin	mouse	Becton Dickinson, HD, G
EpCAM (D5.7)	mouse	ref [1]
ezrin	rabbit	Sigma, Munich, G
FN	mouse	Becton Dickinson, HD, G
GSK3β	mouse	Becton Dickinson, HD, G
HIF1α	mouse	Dianove, Hamburg, G
HSP70	mouse	Becton Dickinson, HD, G
Lamp1	mouse	Stressgen, Canada
LEF	rabbit	Santa Cruz, HD, G

Antibody	Origin	Supplier
MMP2	rabbit	Dianova, Hamburg, G
MMP3	rabbit	Santa Cruz, HD, G
MMP9	rabbit	Dianova, Hamburg, G
MMP13	rabbit	Dianova, Hamburg, G
MMP14	rabbit	Santa Cruz, HD, G
mTOR	rabbit	Santa Cruz, HD, G
Nanog	rabbit	Santa Cruz, HD, G
N-Cadherin	mouse	Becton Dickinson, HD, G
Notch	mouse	Biolegend, San Diego, Ca, US
Oct3/4	rabbit	Santa Cruz, HD, G
p-Akt	mouse	Becton Dickinson, HD, G
p-BAD	mouse	Cell Signaling, Frankfurt, G
p-β-catenin	rabbit	BioTrend, Cologne, G
p-PI3K	mouse	Santa Cruz, HD, G
p-PI3K	rabbit	Cell Signaling, Leiden, NL
p-Pten	rabbit	Cell Signaling, Leiden, NL
Pten	mouse	Becton Dickinson; HD; G

Rab5	rabbit	Santa Cruz, HD, G
Rab7	rabbit	Santa Cruz, HD, G
Rab11	rabbit	Santa Cruz, HD, G
RhoA	mouse	Becton Dickinson
slug	rabbit	Santa Cruz, HD, G
Smac/diablo	mouse	Becton Dickinson; HD; G
snail	rabbit	Santa Cruz, HD, G
SOX2	rabbit	Santa Cruz, HD, G
src	rabbit	Santa Cruz, HD, G
TCF4	mouse	Santa Cruz, HD, G
tubulin	mouse	Becton Dickinson, HD, G
twist	rabbit	Becton Dickinson, HD, G
vimentin	mouse	Becton Dickinson, HD, G
Wnt-1	rabbit	Santa Cruz, HD, G
Wnt5a/b	rabbit	Santa Cruz, HD, G
ZEB-1	rabbit	Santa Cruz, Heidelberg, G
dye or biotin labeled secondary		
antibodies /Streptavidin		Dianova, Becton Dickinson, Amersham

REFERENCES

1. Matzku S, Wenzel A, Liu S, Zöller M. Antigenic differences between metastatic and non-metastatic rat tumor variants characterized by monoclonal antibodies. Cancer Res. 1989; 49:1294–1299.
2. Rana S, Claas C, Kretz CC, Nazarenko I, Zöller M. Activation-induced internalization differs for the tetraspanins CD9 and Tspan8: Impact on tumor cell motility. Int J Biochem Cell Biol. 2011; 43:106–119.
3. Ladwein M, Pape UF, Schmidt DS, Schnölzer M, Fiedler S, Langbein L, Franke WW, Moldenhauer G, Zöller M. The cell-cell adhesion molecule EpCAM interacts directly with the tight junction protein claudin-7. Exp Cell Res. 2005; 309:345–357.

[a]Abbreviations: EAACC: European Association of Animal Cell Cultures, Porton Down, UK.
